# Targeting developmental vulnerabilities in childhood sarcomas

**DOI:** 10.1007/s10555-025-10286-y

**Published:** 2025-09-22

**Authors:** Elise Young, Barnaby Kelly, Jason E. Cain

**Affiliations:** 1 Hudson Institute of Medical Research, 27-31 Wright St, Clayton, Victoria 3168 Australia; 2 Department of Molecular and Translational Medicine, School of Medicine, Nursing and Health Sciences, Monash University, Clayton, Victoria 3800 Australia; 3 Monash Children’s Cancer Centre, Monash Children’s Hospital, Monash Health, Clayton, Victoria 3168 Australia; 4 Department of Paediatrics, Monash University, Clayton, Victoria 3168 Australia

**Keywords:** Childhood cancer, Sarcoma, Signalling, Developmental pathways, Therapeutics

## Abstract

Childhood sarcomas are an aggressive and diverse group of mesenchymal-origin malignancies that collectively account for over a third of paediatric solid tumours. There has been little progress made in the treatment of childhood sarcomas in recent decades, and survival outcomes are poor compared to most other common paediatric cancers. Furthermore, long-term survivors of childhood sarcomas face disproportionately high morbidity from treatment. A unique feature of paediatric and adolescent sarcomas, compared to adult-type sarcomas, is that they arise from developing tissues and often share features with tissue-specific progenitors suggesting that they originate from cells that are arrested in a primitive developmental window. The developmental origins of paediatric sarcomas are also reflected in the incidence of different sarcoma types which correlate with age-specific tissue expansion and growth. In this review, we discuss the molecular mechanisms underpinning paediatric sarcomagenesis, focusing on how distortion of normal developmental programming, such as epigenetic regulation, embryonic signalling pathways, and aberrant growth pathways, contributes to childhood sarcoma development and progression. Finally, we will review the new and emerging therapeutic strategies seeking to target these developmental vulnerabilities.

## Introduction

Childhood sarcomas are a diverse and aggressive group of cancers that originate in the mesenchymal-derived connective tissue of the body and can arise in any anatomical region. Bone and soft tissue sarcomas collectively account for more than a third of paediatric extracranial solid tumours and are the fourth most common type of childhood cancer overall [1]. In recent decades, Many therapeutic advances have improved the survival of childhood cancer to approximately 85% and reduced morbidity from treatment, but sarcomas as a group have not had these benefits [1]. For most childhood sarcomas, survival rates have been unchanged since the 1980s, with an overall survival rate of 73% and 70% for soft tissue and bone sarcomas respectively, which decreases to 10–30% for relapsed, treatment-refractory or metastatic disease [1, 2]. These statistics predominantly reflect the prognosis of the three most common sarcoma types in children and adolescents, which are rhabdomyosarcoma, osteosarcoma and Ewing sarcoma [1, 2]. However, certain rare paediatric sarcomas have even more dismal prognoses such as desmoplastic small round cell tumour (DSRCT) and Malignant rhabdoid tumours which have a 5-year overall survival rate of 16% and 18% respectively [3–5].

Most children and adolescents who survive sarcomas experience long-term and often permanent treatment-related morbidity. Current therapy consists of systemic treatment with multi-agent cytotoxic chemotherapy and aggressive local control techniques utilising surgery and/or radiotherapy. This results in numerous long-term sequelae including infertility, cardiac dysfunction, renal and hearing impairment, secondary malignancies, physical disability and disfigurement, and psychosocial impacts [6]. With a few rare exceptions outlined later, there has been very little progress made in the treatment of childhood sarcoma in recent decades, as is demonstrated by stagnant survival rates [7]. It is evident that new, more effective, specific and safer therapies are urgently needed.

## Developmental origins of childhood sarcomas

**Fig. 1:**
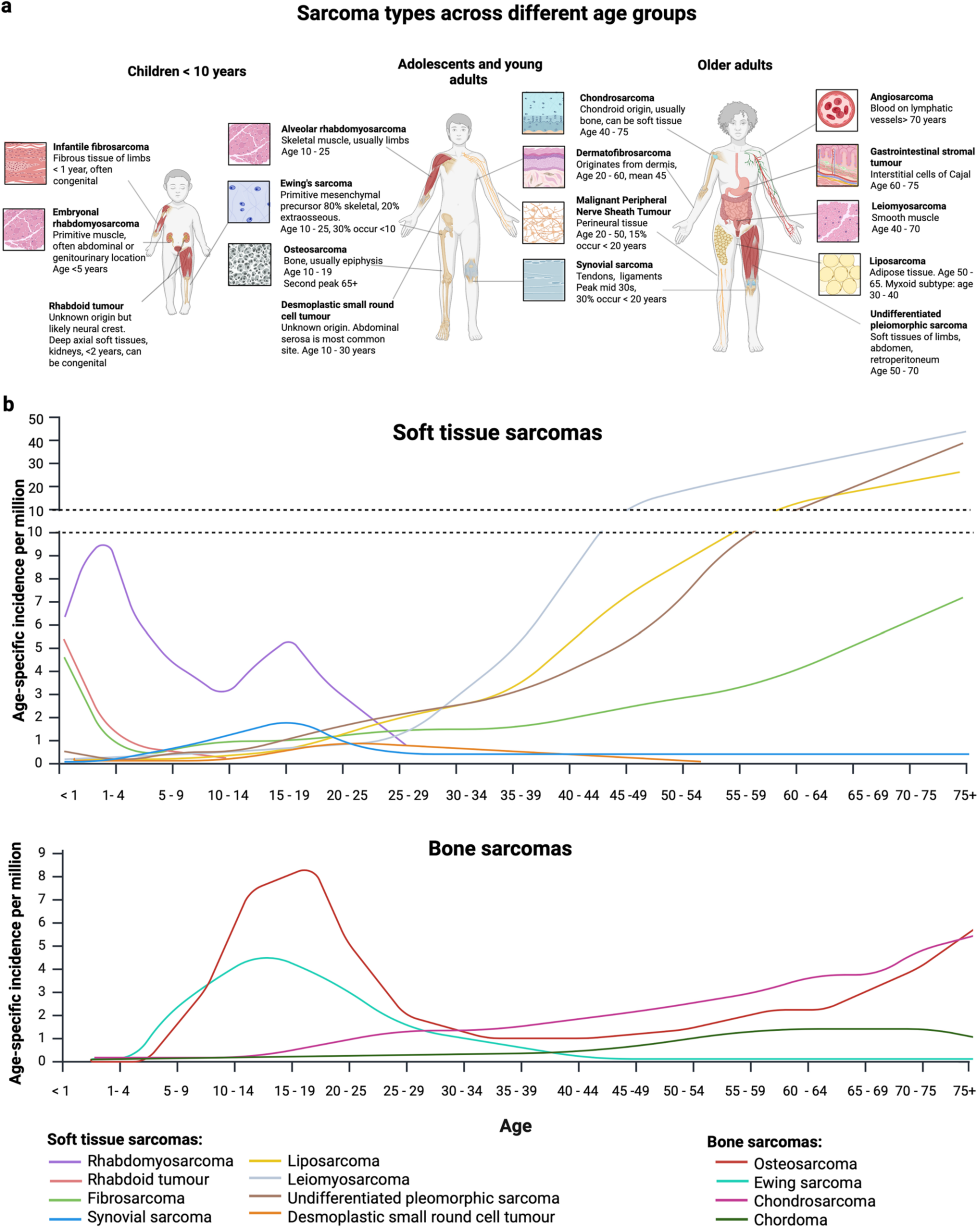
Types of bone and soft tissue sarcoma across the age spectrum. **a** Suspected tissue of origin (or resemblance) in various sarcomas across different age groups. Note that there are varying age definitions of ‘adolescent and young adult’ cancers—the World Health Organisation (WHO) definition of 10–24 years is used here. **b** Age-specific incidence per million of soft tissue and bone sarcomas. Statistics were reproduced from the following sources: non-rhabdomyosarcoma soft tissue sarcomas: SEER cancer statistics 1993–2007 published by Ferrari et al. [11]. Bone sarcomas: National Cancer Intelligence Network data set 1985–2009 published in Beckingsale et al. [10], rhabdomyosarcoma [12], desmoplastic small round cell tumour [13], and extracranial malignant rhabdoid tumour [14]. Note that data on the incidence of malignant rhabdoid tumours and rhabdomyosarcoma were not available beyond age 20. Selected sarcomas, including gastrointestinal stromal tumours and dermatofibrosarcoma, are not shown

A unique feature of paediatric and adolescent sarcomas, compared to adult-type sarcomas, is that they arise from developing tissues and growing evidence suggests they originate from mesenchymal stem or tissue-specific progenitor cells. This is further highlighted by the peak incidence of various sarcoma types during childhood and adolescence, which reflects age-specific development and expansion of the origin tissue [1, 8]. Connective tissue development and turnover can be categorized by the following: (1) modelling, that is primary tissue generation from embryogenesis to adulthood; and (2) remodelling, which can be physiological or pathological and serves to maintain and regenerate tissues in response to ageing, damage or changing physiological conditions [9]. Paediatric sarcoma types predominantly arise from tissues undergoing primary tissue modelling and development (Fig. 1), whereas adult-type sarcomas arise from tissues which undergo remodelling throughout the lifetime [8]. The bimodal distribution of the incidence of osteosarcoma is an excellent example of this. The highest incidence of osteosarcoma occurs between the ages of 15–17 in boys, 14–16 years in girls, correlating with peak primary skeletal growth [10]. However, a smaller secondary peak is seen in older adults over the age of 65, predominately due to excessive bone remodelling disorders such as Paget’s disease of the bone [10].

Developmental windows of various connective tissues also account for the striking differences in cell of origin and thereby specific sarcoma prevalence, between paediatric and adult sarcoma. For example, sarcomas arising from primitive bone or muscle precursors, such as osteosarcoma, rhabdomyosarcoma and Ewing sarcoma, predominate in childhood and adolescence, reflecting primary development and maturation of these tissues, but become rare in adulthood. Comparatively, liposarcoma and chondrosarcoma, which are uncommon in childhood and adolescence, are amongst the most common soft tissue and bone sarcomas in adulthood respectively, arising from tissues that undergo continuous remodelling throughout a lifetime [8]. Similarly, certain types of sarcomas are histologically and molecularly distinct depending on the age at which they arise, again highlighting the differences in developmental origins. For example, embryonal rhabdomyosarcoma, which histologically and molecularly resembles a primitive embryonic rhabdomyoblast, predominantly affects young children [15], whereas the fusion-driven alveolar-type peaks in adolescents and the rare pleomorphic variant arise in older adults (Fig. 1, Table 1).

**Table 1:** Overview of molecular drivers of childhood and young adult sarcoma types

Sarcoma type	Subtype	Peak age	Genetic change	Gene(s)	Frequency of alteration	Ref
Rhabdomyosarcoma	Embryonal	< 5 years	Inactivation or deletion	*CDKN2A/B*	23%	[16, 17]
				*TP53*	5.3%	
				*BCOR*	7.4%	
				*NF1*	10%	
			Activating mutations	*RAS* pathway	20—42%	
				*FGFR4*	9—20%	
				*PIK3CA*	7.4%	
	Alveolar	Adolescents	Fusion	*PAX3::FOXO1*	88%	[18]
				*PAX7::FOXO1*	12%	
	Spindle cell/sclerosing	Broad range	Mutation	*MYOD1 (L122R* mutation)	63%	[19]
		0–3 months	Fusion	*VGLL2 fusions*	63%	
				*NCOA2 fusions*	27%	
	Pleomorphic	> 50 years	Mutation	*TP53*	79%	[20]
				*RB1*	43%	
Osteosarcoma	Conventional/high grade	15 years	Inactivation or deletion	*TP53*	50%	[21–23]
				*RB1*	29–47%	
				*ATRX*	29%	
			Amplification	*c-Myc*	39–42%	
				*VEGFA*	23%	
				*RAD21*	38%	
	Low grade	30 years	Amplification	*12q amp (MDM2 & CDK24)*	89%	[24]
Ewing sarcoma		10–15 years	Fusion	*EWSR1::FLI1*	85–92%	[25, 26]
				*EWSR1::ERG*	5–8%	
				*EWSR1::ETV1*	< 1%	
				*EWSR1::ETV4*	< 1%	
				*EWSR1::FEV*	< 1%	
Sarcoma with BCOR alteration		Adolescents	Fusion	*BCOR::CCNB3*	Common	[27, 28]
				Other *BCOR* fusion	Rare	
	Infantile URCS or PMMTI *	< 1 year	Internal tandem duplication	*BCOR*	85%	
			Fusion	*YWHAE::NUTM2B/E*	15%	
CIC-rearranged sarcoma		25–35 years	Fusion	*CIC::DUX4*	95%	[29, 30]
				Other *CIC* fusion	< 5%	
Synovial sarcoma		Young adults	Fusion	*SS18::SSX1*	61%	[31]
				*SS18::SSX3*	37%	
				*SS18::SSX4*	rare	
Desmoplastic small round cell tumour		10–30 years	Fusion	*EWSR1::WT1*	100%	[26]
Malignant peripheral nerve sheath tumour	Conventional type	20–50 years, 15% occur < 20	Inactivation or deletion	*NF1*	87–100%	[32]
				*CDKN2A*	75%	
				*SUZ12*	56%	
				*TP53*	40%	
				*EED*	32%	
	Epithelioid type		Mutation/deletion	*SMARCB1*	75%	[33]
Alveolar soft part sarcoma		15–35 years	Fusion	*ASPSCR1::TFE3*	100%	[34]
Infantile fibrosarcoma		Congenital—1 year	Fusion	*ETV6::NRTK3*	90%	[35]
				Other *NTRK* fusions	10%	
Malignant rhabdoid tumour		12–18 months	Deletion or inactivation	*SMARCB1*	98%	[36, 37]
				*SMARCA4*	0.5–2%	
Epithelioid sarcoma		10–35 years	Deletion or inactivation	*SMARCB1*	90%	[38]

*Infantile URCS/PMMTI = infantile undifferentiated round cell sarcoma/primitive myxoid mesenchymal tumour of infancy

At a molecular level, paediatric sarcomas are also distinct from their adult counterparts. Like many other childhood cancers, paediatric sarcomas have a lower genomic mutational burden and are more likely to have a simple karyotype and be driven by dysregulated epigenetic programming and transcriptional activity [39]. This reflects less time to accumulate genetic mutations from lifetime exposure to mutagens and aging and challenges the tempo of traditional adult models of tumourigenesis [39]. Simple-karyotype sarcomas are driven by a single genomic aberration, and although they can accumulate other mutations over time, they have relatively stable genomes [39, 40]. Often, the driving genomic event is a fusion gene that encodes a chimeric transcription factor which catastrophically alters transcription and epigenetic regulation, such as in Ewing sarcoma, alveolar rhabdomyosarcoma, synovial sarcoma, or DSRCT [41] (Table 1). However, in some cases, the genomic driver can be a different event, such as in malignant rhabdoid tumours which are exclusively driven by biallelic inactivation of the tumour suppressor genes SWI/SNF related Matrix associated, actin dependent regulator of chromatin, subfamily b, member 1 (*SMARCB1*) or less frequently *SMARCA4*, both of which encode critical subunits of the Switch/Sucrose Non-Fermentable (SWI/SNF) chromatin remodelling complex that regulate large sets of genes required for key developmental processes [36]. Contrastingly, complex-karyotype sarcomas are chromosomally unstable, leading to the accumulation of numerous aneuploidies. Mutations in genes involved in DNA-damage response and apoptosis such as *TP53* and *RB1* are common in these types of sarcomas, which allow cells to resist apoptosis despite dysregulated genomes (Table 1). Osteosarcoma is the cardinal example of a complex-karyotype paediatric and adolescent sarcoma, where chromothripsis due to severe chromosomal instability is commonly seen in high-grade tumours [21].

Many paediatric sarcomas have a histological and transcriptomic resemblance to embryological tissues or developmental progenitor cells, suggesting that they arise from cells that became arrested in a primitive developmental window because of a ‘differentiation block’. However, this dogma has recently been challenged by the concept of transdifferentiation, also termed lineage reprogramming [42, 43]. There are several observations that support the notion that tissue of resemblance or differentiation of sarcomas may not represent the true tissue of origin. For example, rhabdomyosarcoma can arise in tissues that are completely devoid of skeletal muscle, Ewing sarcoma can arise in almost any extra-osseus location including even the brain or viscera, and although synovial sarcoma has some morphological resemblance to synovial tissue, it does not usually arise in the synovium [44, 45]. These observations could be explained by either (1) transdifferentiation, a process by which one differentiated tissue can be converted to another without going through an intermediate stem cell stage, or (2) that these cancers arise from primitive stem cells which then aberrantly express features of differentiated tissues that would not usually occur in that location. Intriguingly, recent progress with *in vivo* models has provided much insight into this. A number of murine models of rhabdomyosarcoma have demonstrated substantial developmental heterogeneity. Both endothelial progenitors and adipocytes have been demonstrated to give rise to tumours which histologically and molecularly resemble rhabdomyosarcoma through myogenetic reprogramming [43] and are further reviewed by Stevens et al. [44]. Similarly, in a novel zebrafish model of Ewing sarcoma, targeted expression of EWSR1::FLI was uniquely tolerated in the neural crest and resulted in mesodermal reprogramming [46].

## Epigenetic dysregulation

Epigenetic programming controls gene expression without altering the genetic code and is a fundamental process underpinning embryogenesis and foetal development. Through precise regulation of gene expression in a stage-specific and sequential manner, epigenetic regulation ensures that critical processes such as cell specification, cell division and differentiation occur at the appropriate location and time across developing tissues. However, if the tight constraints regulating spatiotemporal gene expression are lost, the same processes that regulate proliferation and differentiation in developing tissues can drive oncogenesis. Epigenetic dysregulation is a prominent mechanism underpinning the development and progression of paediatric cancers. Highlighting this are two recent pan-cancer analyses that identified mutations in genes coding for epigenetic regulators as the most common group of alterations in paediatric malignancies [39, 47]. Furthermore, many of these mutations associated with epigenetic dysregulation are unique to specific cancer types, implicating the temporal, lineage and cell-specific susceptibility and impact on developmental state [47].

**Fig. 2:**
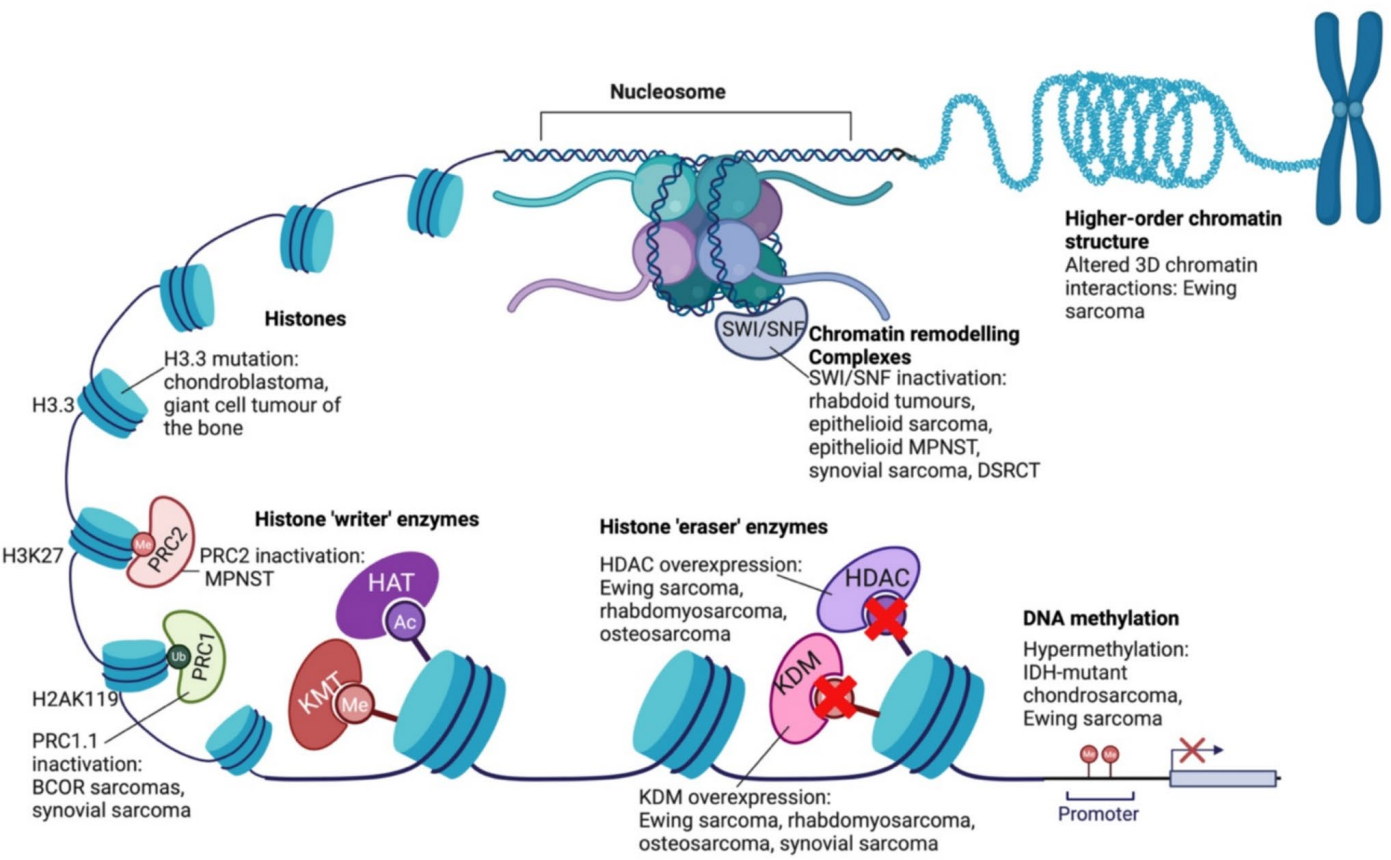
Epigenic mechanisms implicated in childhood sarcomagenesis. Epigenetic regulation is altered through numerous mechanisms in childhood sarcomas. MPNST—malignant peripheral nerve sheath tumour. DSRCT—desmoplastic small round cell tumour. HDAC—histone deacetylase. KDM—histone lysine demethylase. KMT—histone lysine methyltransferase. HAT—histone acetyltransferase. PRC1 and PRC2—polycomb repressor complexes 1 and 2

Epigenetic dysregulation is a particularly prominent feature of many childhood sarcomas and can occur through a variety of different mechanisms. These mechanisms disrupt key components of the epigenetic machinery, including chromatin remodelling complexes, histones, histone-modifying enzymes, or higher-order chromatin organisation and architecture (Fig. 2, Table 2).

**Table 2:** Epigenetic alterations and associated therapeutics in childhood sarcomas

Epigenetic component	Gene	Examples in childhood sarcomas	Function	Potential therapy	Ref
Chromatin remodelling complexes	*SMARCB1*	Malignant rhabdoid tumours, epithelioid sarcoma, epithelioid MPNST, poorly differentiated chordoma, synovial sarcoma	Component of SWI/SNF complex which mobilizes nucleosomes to modulate gene expression	Area of need. No direct inhibitors however SMARCB1-deficient tumours appear dependent on aberrant EZH2 function (see below)	[37, 48]
	*SMARCA4*	Malignant rhabdoid tumours			
	*ATRX*	20–30% of osteosarcoma			
	*ARID1A*	DSRCT			
Writer enzymes	*EZH2*	Dysregulated in SMARCB1-deficient tumours	Aberrant function of PRC2	EZH2 inhibitors - Tazemetostat* - Valemetostat	[49]
	*SUZ12*	Mutated in MPNST	Loss of PRC2 function leading to loss H3K27 trimethylation	Area of need	[50]
	*EED*				
	*BCOR*	BCOR-altered sarcomas	Component of PRC1.1, which ubiquitinates H2AK119	Area of need	[28]
		Dysregulation by SS18-SSX fusion in synovial sarcoma			
	*DNMTs*	Dysregulated in many sarcomas including osteosarcoma, rhabdomyosarcoma	DNA methyl transferase	DNMTs inhibitors: - Decitabine* - Azacitidine*	[51, 52]
Eraser enzymes	*IDH1 or 2*	Mutated in 60% chondrosarcoma, 6% of Ewing sarcoma	Mutant protein produces 2-HG which inhibits DNA and histone demethylases	IDH inhibitors - Ivosidenib - Enasidinib	[53]
	*HDACs*	Overexpression in rhabdomyosarcoma, osteosarcoma, other sarcoma types	Histone deacetylases	HDC inhibitors - Panobinostat* - Romdepsin* - Belinostat* - Vorinostat*	[54, 55]
	*KDM1A*	Ewing sarcoma, osteosarcoma, rhabdomyosarcoma	Histone demethylases	LSD1/KDM1 inhibitors - Ladademstat - Seclidemstat	[56]
Histone mutations	*H3F3A*	Giant cell tumour of the bone**	Regulates gene expression and DNA accessibility	Area of need	[57]
	*H3.3K36*	Chondroblastoma**			
Chromatin structure	*EWSR1* fusions	Ewing sarcoma and related FET-fusion-driven sarcomas	Alters 3D chromatin architecture, transcriptional regulation	EWSR1::FLI inhibitors - TK-216	[58]
	*STAG2*	Ewing sarcoma			[59]

* indicates a therapy that is FDA-approved for another cancer type

** denotes an intermediate grade but locally aggressive mesenchymal tumour. These are included for completeness but not considered a true sarcoma

### Chromatin remodelling complexes

Chromatin is a highly organised condensed structure formed by DNA wrapping around histones to form the nucleosome. This process serves not only to compact the 6 billion base pairs of the diploid human genome into the nucleus of a cell but also plays a pivotal role in regulating gene expression. DNA that is contained within nucleosomes generally cannot be accessed by transcription factors and associated genes are transcriptionally silent [60]. Chromatin remodelling is a process where nucleosomes are mobilised to rearrange chromatin into a transcriptionally accessible state through an intricate network of cellular machinery termed chromatin remodelling complexes.

The SWI/SNF complex is a powerful chromatin remodelling complex that utilizes ATP hydrolysis to reposition nucleosomes, resulting in regulation of proliferation and differentiation in developing tissues [60]. The SWI/SNF complex has tumour-suppressive functions in healthy tissues, and aberrant functions of the SWI/SNF complex are directly associated with oncogenesis in a variety of cancers, most notably, malignant rhabdoid tumours [36]. Malignant rhabdoid tumours are extremely aggressive paediatric malignancies that occur primarily in young children and infants with an average age of onset 12–18 months. While the cell of origin is somewhat undefined, there is strong evidence suggesting it is likely Schwann cell progenitors derived from the neural crest [61]. This would account for their propensity for the soft tissues and the occurrence in the kidneys (malignant rhabdoid tumour of the kidney) and brain (atypical teratoid/rhabdoid tumour).

Malignant rhabdoid tumours are genetically defined by inactivation of the *SMARCB1* gene in almost all cases [37]. The remaining 0.5–2% of cases have a mutation in *SMARCA4* [37]*. SMARCB1* and *SMARCA4* both encode for key subunits of the SWI/SNF complex. The bi-allelic inactivation or deletion of these genes results in dysregulation of cell cycle control, differentiation and apoptosis and activation of numerous oncogenic signalling pathways [36].

*SMARCB1* loss of function is also reported in other sarcomas that affect children and young people, including synovial sarcoma, the epithelioid variant of the malignant peripheral nerve sheath tumour (MPNST), epithelioid sarcoma, and the paediatric subtype of chordoma, poorly differentiated chordoma [37]. The loss of *SMARCB1* in these tumours may occur due to gene alterations, or in the case of synovial sarcoma, due to degradation of *SMARCB1* protein by the *SS18::SSX* fusion transcript [62]. Mutations in other genes encoding key components of the SWI/SNF complex have also been reported in various sarcomas, such as *ATRX* mutations in 30% of osteosarcoma, and *ARID1A* which is recurrently mutated in DSRCT [48, 63].

### Histone mutations

Histones regulate chromatin structure, gene expression and silencing, and ultimately govern normal transcriptional regulation and developmental programming. Histone mutations and modifications are implicated in oncogenesis of numerous cancers. The nucleosome is formed through an octomer of histone proteins, consisting of a tetramer of histones H3 and H4 and 2 dimers of H2A and H2B. H3 is the most frequently mutated histone in cancer, and it is termed the ‘oncohistone’ [64]. Giant cell tumour of the bone and chondroblastoma, which are both benign but locally aggressive bone tumours affecting young people, are primarily driven by H3.3 G34W and H3.3 K36M mutations respectively [57]. The resultant epigenetic dysregulation of genes from these histone mutations affects differentiation and proliferation and ultimately drives tumour development in the primitive bone and cartilage precursors [65, 66].

### Histone modifications

Histone modifying enzymes catalyse the addition or removal of various modifiers, predominantly methyl groups and acetyl groups, onto histone lysine or arginine tails to modify chromatin states. Acetylation results in open, transcriptionally accessible chromatin states, whereas methylation generally condenses and transcriptionally silences chromatin but can also activate transcription depending on the specific histone residue involved [64]. Histone modifying enzymes are further classified as ‘writer’ enzymes including histone lysine (K) methyltransferases (KMTs) and histone acetyltransferases (HATs) or ‘eraser’ enzymes such as histone lysine demethylases (KDMs) or histone deacetylases (HDACs) (Fig. 2) [64].

Histone deacetylases act predominantly as transcriptional repressors of key tumour suppressor genes in cancer and are overexpressed in many sarcoma types including osteosarcoma, liposarcoma, and rhabdomyosarcoma [54]. Functional studies utilising overexpression and knockout approaches have demonstrated that HDAC expression results in more aggressive disease and metastatic qualities in rhabdomyosarcoma and osteosarcoma cell lines [55]. HDACs have been identified through CRISPR screening techniques as a functional dependency for rhabdomyosarcoma proliferation and knockout of *HDAC3* results in differentiation [67]. Furthermore, in zebrafish and murine xenograft models of rhabdomyosarcoma, treatment with HDAC inhibitors recapitulates the HDAC-loss-of-function phenotype with reduced cancer stemness and self-renewal [68].

Histone demethylases such as KDM1A (LSD1) are also overexpressed in many childhood sarcoma types, resulting in loss of methylation at H3K4 and transcriptional activation of oncogenic signalling. In a methylation analysis of 500 sarcomas, KDM1A was overexpressed in synovial sarcoma, rhabdomyosarcoma, osteosarcoma and Ewing sarcoma, and elevated expression is associated with a poorer prognosis in Ewing sarcoma [69, 70]. Furthermore, isocitrate dehydrogenase (IDH) mutations occur in 60% of chondrosarcoma and in 6.5% of Ewing sarcoma. While not a direct histone-modifier itself, IDH mutations result in the production of 2-hydroxyglutarate (2HG) which inhibits demethylation and results in hypermethylation of histones and DNA [53].

Polycomb repressor complexes 1 and 2 (PRC1 and PRC2) interact with histones and chromatin primarily to repress embryonic chromatin landscapes during normal tissue development to maintain differentiation and lineage fidelity. PRC1 achieves chromatin compaction and transcriptional repression through ubiquitinating H2A at lysine 119 (Fig. 2) [71]. BCL6 corepressor (BCOR) is an evolutionarily conserved regulator of trophoblast development and stem cell pluripotency and forms a critical component of PRC1.1 [41, 72]. In BCOR-altered bone and soft tissue sarcomas, the fusion protein interrupts normal function of PRC1, leading to de-repression of the embryonic program, resulting in dysregulated differentiation and proliferation [72–74]. Furthermore, functional genomic studies have identified histone demethylase KDM2B, which is another core component of PRC1.1, as being selectively required for sustaining synovial sarcoma cell transformation [75]. The oncoprotein produced by the SS18::SSX1 fusion aberrantly activates the expression of the developmental program via hijacking the repressive function of PRC1.1 through KDM2B [75].

Polycomb repressor complex 2 (PRC2) suppresses embryonic chromatin landscapes through methylation of H3 at lysine 27 (Fig. 2). PRC2 is comprised of suppressor of zeste 12 (SUZ12), embryonic ectoderm development (EED) and enhancer of zeste homolog 1 and 2 (EZH1 and EZH2) [71]. *SUZ12* and *EED* mutations occur frequently in high-grade MPNST, resulting in inactivation of PRC2 and thus loss of H3K27 trimethylation, which ultimately leads to loss of repression of the embryonic program [50]. Furthermore, EZH2 appears to be dysregulated and aberrantly expressed in *SMARCB1*-deficient tumours, suggesting it may act as a druggable dependency [49]. Finally, STAG2 loss of function occurs recurrently in Ewing sarcoma and disrupts the repressive function of PRC2, leading to expression of developmental genes and induces a more aggressive and metastatic phenotype in murine models [76].

### Complex chromatin interactions and higher-order chromatin structure

Translocation-associated sarcomas often are driven by a chimeric transcription factor that alters gene expression through numerous complex interactions with chromatin occupancy and dynamics, which ultimately switches on a developmental program within a cell, promoting proliferation and disrupting differentiation [41]. The *EWSR1* gene encodes a multifunctional protein that plays a role in diverse cellular processes including RNA binding and regulation of stem cell differentiation through multiple epigenetic pathways [58]. When the N-terminal activating domain of the *EWSR1* protein is fused with the C-terminal of a transcription factor partner such as *FLI1*, it creates an oncogenic chimeric transcription factor that dysregulates the expression of numerous developmental genes through DNA binding and interactions with other transcription factors and transcriptional machinery [41]. This ultimately shifts this cell towards an immature and undifferentiated state [41]. For example, RNA helicase A, a critical regulator of normal ectoderm development, is one of the many transcriptional regulators that is hijacked by EWSR1::FLI [77–79]. Binding of EWSR1::FLI with RNA helicase A has been shown to enhance the oncogenic effect of the fusion through transcriptional coactivation [78, 79]. Furthermore, SOX6, a developmental transcription factor that drives the proliferation of osteo-chondrogenic progenitors, is hijacked by EWSR1::FLI through binding to intronic GGAA-microsatellites resulting in aberrant activation and promoting proliferation of Ewing cells.

Similarly, the fusion of *CIC* with *DUX4* in CIC-rearranged sarcomas converts the transcriptional repressor CIC into a transcriptional activator [80]. In fusion-positive rhabdomyosarcoma, the DNA-binding capabilities of *PAX3* or *PAX7* are fused with the transcriptional activating domain of *FOXO1* [81]. Chromatin occupancy profiling in these fusion-driven sarcomas reveals that these chimeric transcription factors interrupt chromatin dynamics through multiple mechanisms to drive oncogenic programs [80, 81].

Higher-order chromatin architecture refers to the assemblage of nucleosomes into compact 3D structures, which influences gene expression through multiple mechanisms such as promoter-enhancer interactions and adds another layer to DNA accessibility. The dynamics of higher-order chromatin structure in sarcomas is still relatively poorly understood, but emerging evidence suggests various fusion genes that occur in Ewing and related sarcomas mediate genome-wide changes in chromatin configuration and 3D structure [58]. Furthermore, loss of function mutations in *STAG2* occurs in 17% of Ewing sarcoma and is associated with a more aggressive tumour phenotype both in clinical studies and in murine xenograft models [59, 76]. STAG2 is part of the cohesin complex which regulates chromatin organisation. Loss of STAG2 function in Ewing sarcoma cells alters chromatin architecture and, in combination with the disruption of PRC2, activates an oncogenic developmental program to promote migration and metastasis [59, 76].

### Targeting epigenetic dysregulation

The frequency of epigenetic alterations implicated in childhood sarcomagenesis provides a strong rationale for the therapeutic potential of epigenetic modifying drugs. Histone deacetylase inhibitors (HDACi) have shown success in treating some haematological malignancies and are now FDA-approved for this indication [64] and are currently under investigation in epigenetically driven paediatric sarcomas. In orthotopic xenograft models of rhabdoid tumours, the pan-HDACi panobinostat inhibited tumour growth, extended survival and drove differentiation of tumour cells [82]. In osteosarcoma, HDACi reduced primary tumour growth [83] and prevented lung metastasis in mice inoculated with osteosarcoma cells via tail vein injection [84]. HDACi have also been shown to radiosensitise rhabdomyosarcoma cell lines [85]. Furthermore, in rhabdomyosarcoma xenograft models, the HDACi mocetinostat showed synergistic activity with vinorelbine, a mitotic inhibitor, and is now in phase I trial for adolescents > 12 years (MCT04299113). Preliminary findings reported that four of eight participants had a partial response and two had stable disease. A phase II study of panobinostat in paediatric, adolescent and young adult patients with neuroblastoma, osteosarcoma or malignant rhabdoid tumours (ACTRN12618000321246) recently completed 2-year follow-up, and results are pending.

SP-2577 (seclidemstat), a selective inhibitor of KDM1A, demonstrated *in vivo* efficacy in a variety of paediatric sarcomas [56]. It is currently in phase I/II clinical trials in *EWSR1*-fusion sarcomas (NCT03600649) including Ewing sarcoma, myxoid liposarcoma, and DSRCT.

In a phase II trial of EZH2-inhibitor tazemetostat for people > 16 years with *SMARCB1*-deficient advanced epithelioid sarcoma, 15% had an objective response and the median progression-free survival was 5.5 months, prompting FDA approval [86]. In pre-clinical models of malignant rhabdoid tumours, tazemetostat demonstrated significantly prolonged time to endpoint but did not induce tumour regression [87]. A phase II study of tazemetostat in 20 children with *SMARCB1*-deficient tumours, including malignant rhabdoid tumours, failed to meet primary endpoint with an objective response rate in only 1 child, but 6-month progression-free survival was 35%, suggesting it may have a modest effect on disease stabilisation [88].

Targeting chimeric transcription factors is of great interest, given they are specific to malignant tissue and are the main oncogenic driver in fusion-driven sarcomas. However, given the diverse and complex functions and the lack of drug binding sites, such as enzymes or receptors, transcription factors have traditionally been considered undruggable targets. More recently, it has been demonstrated that small molecule inhibitors can disrupt the interaction between an oncogenic transcription factor and a key binding partner to reduce its oncogenic effects. In Ewing sarcoma, small molecule YK-4-279 and its clinical derivative TK-216 have been labelled as first-in-class inhibitors of EWSR1::FLI1 and other EWSR1 fusions seen in Ewing sarcoma. YK-4-279 and TK-216 bind to EWSR1::FLI1, preventing it from interacting with other transcription proteins, in particular RNA helicase A. YK-4-279 induced apoptosis *in vitro* and substantially inhibited tumour growth in orthotopic mouse xenograft models [78] but acquired resistance has been observed [89]. A phase I/II trial of TK-216 in patients with relapsed or refractory Ewing sarcoma enrolled 48 patients in the phase II arm who were treated with the recommended phase II dose of TK-216, in addition to vincristine in 46 of the 48 patients [90]. There were only 3 objective responses reported (6%), including 1 partial response and 2 complete responses. The 2 patients with complete response had a remarkable and sustained remission of 28 months and 46 months respectively [90].

## Developmental signalling pathways: balancing proliferation and differentiation

The Hedgehog (Hh), Notch, WNT/β-catenin, and Hippo signalling pathways are among the most studied and important mammalian embryonic signalling pathways. These pathways are critical regulators of differentiation and key determinants of tissue lineage commitment of precursor cells, required to generate healthy tissue and normal organ development. Dysregulation of these pathways leads to an imbalance of differentiation and proliferation and is associated with developmental abnormalities and cancer, including childhood sarcomas.

### Hedgehog signalling

**Fig. 3:**
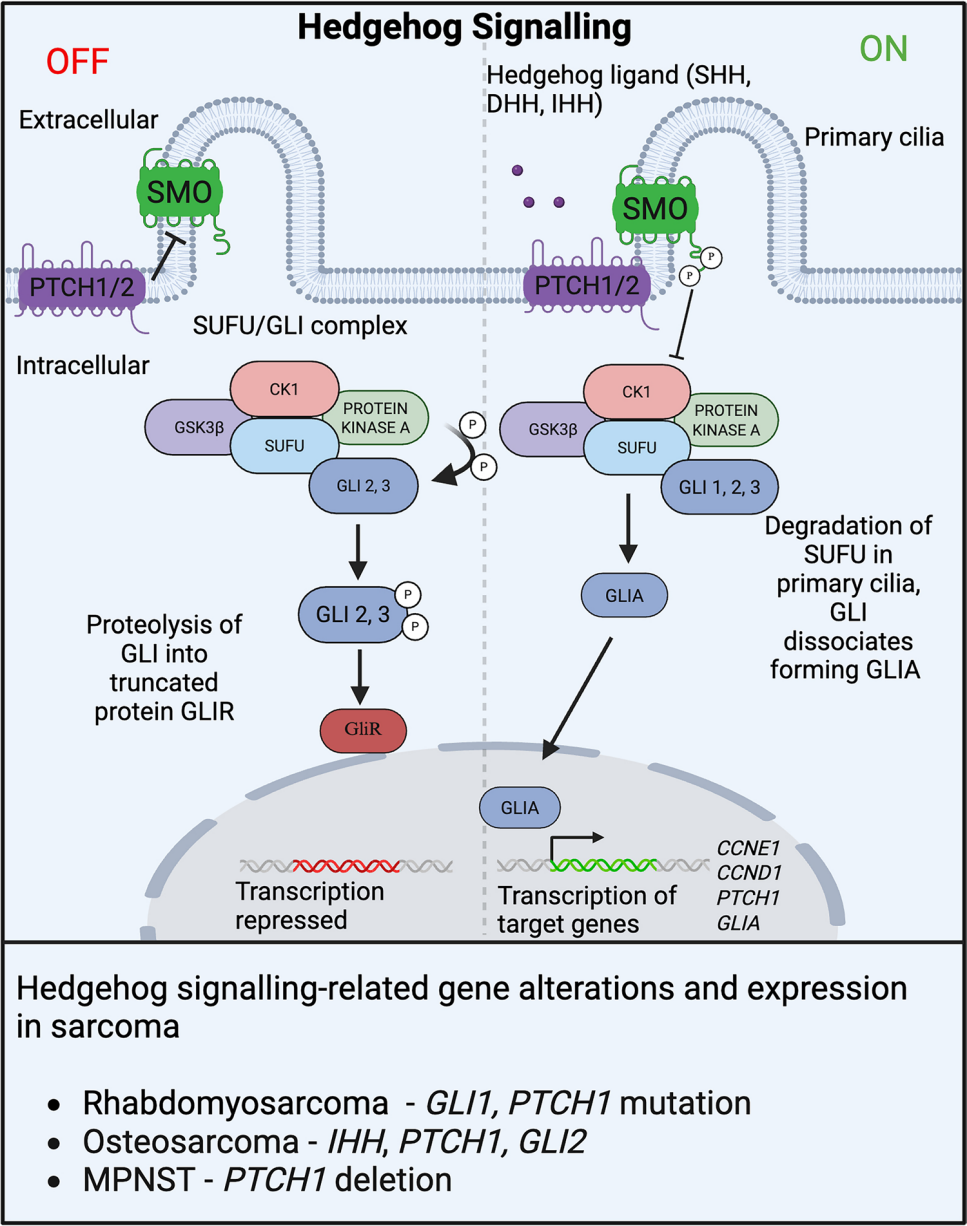
Hedgehog signalling in childhood sarcoma. Overview of Hh signalling pathway in sarcoma. Hh signalling relies on the binding of external ligands to transmembrane PTCH proteins, that in turn stimulates the activation of SMO. SMO activation leads to inhibition of the SUFU/GLI complex (SUFU, GSK3β, PKA, CK1 and GLI) preventing GLI phosphorylation ultimately triggering SUFU/GLI complex degradation in the primary cilia. GLI proteins are then stabilized, resulting in their active form GLIA, whereby upon entering the nucleus, they drive transcription of Hh target genes *CCNE1*,* CCND1*,* PTCH1* and GLI1. In the absence of ligand, GLI phosphorylation by PKA results in proteolytic degradation into a truncated product that represses gene transcription

Hh signalling is an evolutionarily conserved developmental pathway that is tightly regulated in a spatial and temporal manner for normal cell fate determination, self-renewal and tissue patterning [91]. Mammalian canonical Hh signalling activation is driven by one of three Hh ligands, Sonic Hedgehog (SHH), Indian Hedgehog (IHH) and Desert Hedgehog (DHH). In the absence of Hh Ligands, transmembrane proteins Patched 1 (PTCH1), and to a lesser extent Patched 2 (PTCH2), constitutively repress the G protein-coupled-like receptor Smoothened (SMO) [92]. In this state, full-length Glioma-associated oncogene family of latent zinc-finger transcriptional factor (GLI) proteins (GLI2, GLI3) form a complex with Suppressor of fused (SUFU), Glycogen synthase kinase-3 (GSK3β), Protein kinase A (PKA) and Casein kinase 1 (CK1). PKA phosphorylates GLI2 and GLI3 which are then constitutively processed into their transcriptional GLI repressor (GLIR) forms (Fig. 3). In the presence of ligand, PTCH1/2 is internalised and degraded, removing its inhibitory block on SMO allowing SMO phosphorylation. Activated SMO inhibits PKA activity, preventing phosphorylation of GLI leading to the degradation of the SUFU complex within the primary cilia [93]. Full-length GLI1,2,3 is then phosphorylated at a cluster of partial consensus PKA sites (Pc-g) [94] enabling nuclear translocation of the GLI activator (GLIA) form and activation of transcription for target genes including *CCNE1*, *CCND1*, *PTCH1* and *GLI1* [95] (Fig. 3).

A role for aberrant Hh signalling in sarcomagenesis was first realised in patients with Gorlin’s syndrome, caused by inactivation of the *PTCH1* or *SUFU* genes, who are predisposed to the development of basal cell carcinomas, medulloblastoma, fibrosarcoma and rhabdomyosarcoma [96–98]. Approximately a third of embryonal rhabdomyosarcomas have a loss of chromosome region 9q22 including the *PTCH1* locus, leading to constitutive Hh pathway activation [99]. Amplification of the chromosome region 12q13-15, which includes *GLI1*, is also reported in embryonal rhabdomyosarcoma [17]. Furthermore, embryonal and alveolar rhabdomyosarcoma frequently have increased expression of *PTCH1* and GLI1, transcriptional targets of the Hh signalling pathway, and increased Hh pathway activation is associated with rapid progression and poor outcomes [100]. These data are supported by preclinical models, with tumours histologically consistent with embryonal rhabdomyosarcoma developing in 10% of *Ptch1*^±^ mice on the CD1 genetic background and also in mice with a SMO point mutation (*SmoM2*–*W535L*) leading to constitutive Hh pathway activation in non-myogenic endothelial progenitor cells, resulting in a block in myogenesis and driving tumorigenesis [101, 102]. Targeting Hh pathway activation in rhabdomyosarcoma using small molecular SMO inhibitors, vismodegib, sonidegib and saridegib, has been demonstrated to inhibit growth in embryonal rhabdomyosarcoma cell lines [103].

Expression of Hh pathway signalling components and transcriptional target genes such as *SHH*, IHH, *PTCH1*, *SMO*, and *GLI2* is also associated with high-grade osteosarcoma and poor outcomes [95, 104, 105]. The functional importance of Hh signalling in osteosarcoma is highlighted in preclinical studies in which pathway activation is inhibited at the level of SMO or GLI, leading to reduced osteosarcoma cell growth *in vitro* and tumour growth in *in vivo* xenograft models [95, 104]. In contrast to rhabdomyosarcoma, genetic alterations in Hh pathway members leading to constitutive pathway activation are not observed in osteosarcoma, and pathway activation is instead ligand dependent. Genetic alterations of *TP53* and *RB1* are common in osteosarcoma. The loss of normal function of these genes during development and in osteosarcoma leads to dysregulated autophagy, aberrant cilia formation and hypersensitivity to Hh ligand, proposing a mechanism of aberrant Hh pathway activation [105]. Inhibition of Hh signalling using sonidegib in TP53-deficient allograft and xenograft models, or genetic inactivation of *Smo* in the *Trp53* and *Rb1* conditional genetic mouse osteosarcoma model, leads to tumour inhibition and osteosarcoma cell differentiation into mature bone, suggesting that Hh signalling acts to prevent terminal differentiation, enabling tumour growth [105]. Despite promising results in osteosarcoma preclinical models, targeting the Hh pathway in osteosarcoma patients has not yet successfully translated into a clinical setting. In a phase I trial of sonidegib in children with locally advanced solid tumours, a small cohort of five osteosarcoma patients failed to respond to treatment [106]. However, it is important to note that none of these patients exhibited a Hh pathway activation signature. Interestingly, although the safety profile of sonidegib in children was similar to adults, premature growth plate closure was observed in some children and represents an on-target effect of Hh inhibition and poses a limiting factor in treating children and young adolescents with Hh pathway inhibitors [106].

Dysregulated Hh signalling is also observed in MPNST. Multiplatform molecular profiling of 108 tumours identified two distinct subsets with different drivers of progression: one related to Hh signalling (MPNST-G1) and the other to WNT/β-catenin (MPNST-G2) [107]. The MPNST-G1 subpopulation was found to have a lower relapse-free survival rate than the G2 counterpart and harboured deletions in *PTCH1*, leading to constitutive Hh pathway activation [107].

### Notch signalling

**Fig. 4:**
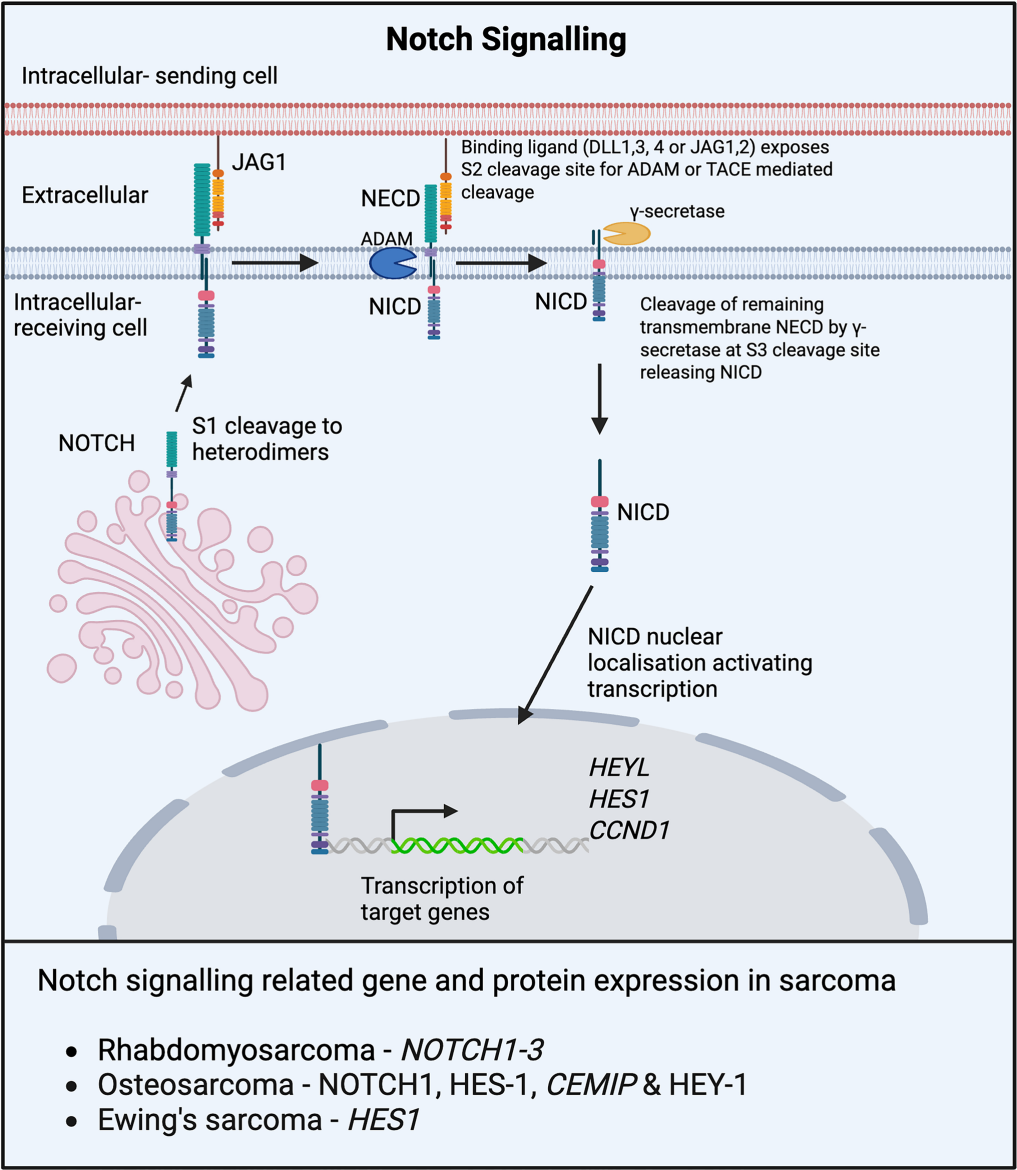
Notch signalling and its dysregulation in childhood sarcoma. Overview of the notch signalling pathway. Notch signalling relies on three sequential cleavage steps. S1 cleavage: glycosylation of NOTCH enabling cleavage in the Golgi apparatus followed by membrane localisation. S2 cleavage: now transmembrane, NOTCH interacts with ligands such as JAGGED, enabling the cleavage by metalloproteases such as ADAM10, removing NECD. S3: Cleavage at S2 exposes the S3 site, which is cleaved by γ-secretase, releasing NICD from the membrane, where it then travels to the nucleus to initiate the transcription of target genes *HEYL*, *HES1* and *CCND1*

Notch signalling regulates cell fate, tissue development, differentiation, proliferation and homeostasis through direct cell-to-cell contact. Maturation of Notch receptors (NOTCH1-4) occurs in the Golgi complex and involves cleavage at the S1 site of the prospective extracellular side of the receptor [108]. Cleaved NOTCH heterodimers translocate to the plasma membrane becoming transmembrane and bind one of five ligands (Jagged: JAG1, JAG2 and Delta-like: DLL1, DLL3, DLL4) stimulating allosteric conformation changes allowing for cleavage at a conserved S2 site by metalloproteases such as A Disintegrin and Metalloproteinase 10 (ADAM10), which releases the extracellular component of the receptor, NOTCH extracellular domain (NECD) [109] (Fig. 4). With NECD removed, the S3 site becomes accessible and is cleaved by γ-secretase, releasing the NOTCH intracellular domain (NICD), which translocates into the nucleus to stimulate the transcription of target genes such as *HEYL*, *HES1* and *CCND1* [110] (Fig. 4). Dysregulation of Notch signalling is strongly implicated in sarcomagenesis, particularly in rhabdomyosarcoma, osteosarcoma, and Ewing sarcoma [111].

In rhabdomyosarcoma patient tissues and preclinical models, overexpression of *NOTCH1*−3, compared to normal muscle, is accompanied by increased expression of the transcriptional targets *HES1* and *HEY1* and is associated with increases in proliferation, migration, invasion and prevention of differentiation [112, 113]. Highlighting Notch-mediated maintenance of an undifferentiated state in rhabdomyosarcoma, pathway inhibition using a γ-secretase inhibitor or direct inhibition of NOTCH3, HEY1 or HES1 increases the expression of myogenic differentiation factors and drives muscle-like differentiation [111, 113].

Increased expression of NOTCH1, NOTCH3, HES1 and HEY1 is also observed in pre- and post-treatment osteosarcoma patient samples with NOTCH3 expression associated with poorer 5-year survival [114]. Notch signalling in osteosarcoma can be indirectly activated by cell migration-inducing protein (CEMIP) that is overexpressed in osteosarcoma patients and associated with poor prognosis. Inhibition of CEMIP function reduces Notch signalling activation and suppresses osteosarcoma growth and metastasis *in vitro* and *in vivo *[115]. The development of osteosarcoma-like tumours in a genetically engineered mouse model expressing NCID in immature osteoblasts further highlights the contribution of primitive osteoblast cells in the development of osteosarcoma [116].

The EWSR1::FLI1 fusion has been shown to increase transcription of *MFNG* [117], a glycosyltransferase that regulates S2 cleavage of NOTCH receptors through enhancing DLL1 binding, reducing the binding of alternative ligands such as JAGGED1 [118]. Interestingly, inhibition of Notch signalling in Ewing sarcoma preclinical models induces neuroectodermal differentiation but has no effect on proliferation of tumour growth suggesting a primary role of maintaining an undifferentiated phenotype rather than driving proliferation [119].

### WNT-β catenin signalling

The WNT-β catenin signalling pathway plays critical roles in embryonic development and tissue homeostasis, including regulating the proliferation and differentiation of mesenchymal stem cells and their derivative tissues such as cartilage and synovium [120]. In cancer, WNT signalling drives tumour development, metastasis, tumour progression, angiogenesis and modulation of the tumour microenvironment and is implicated in bone and soft tissue sarcomas [121].

**Fig. 5:**
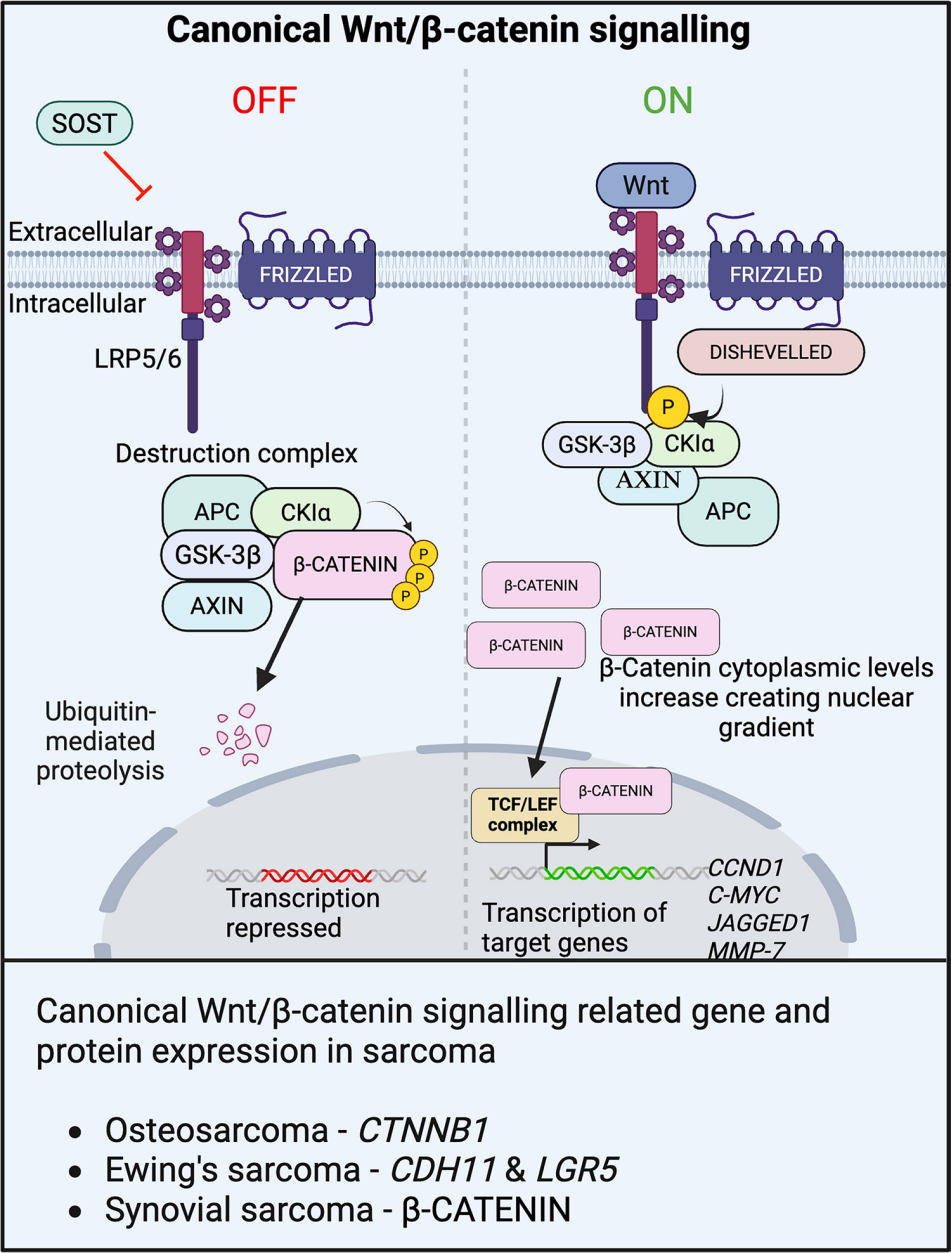
Wnt/β-catenin signalling pathway and its dysregulation in childhood sarcoma. Overview of canonical Wnt/β-catenin signalling. In the absence of WNT or in the presence of antagonist SOST, FZD and LRP5/6 are unable to recruit the β-catenin destruction complex, leading to its degradation via proteolysis. In the presence of WNT, active FZD-LRP5/6 recruit Dishevelled (DVL) preventing β-catenin phosphorylation and promoting β-catenin dissociation with the destruction complex raising cytoplasmic concentrations of β-catenin allowing for nuclear translocation and transcription of target genes following binding to the promoter region and the TCF/LEF complex

The WNT signalling pathway is separated into two forms: β-catenin dependent (canonical) and β-catenin independent (noncanonical). Here, we will focus on the canonical WNT/β-catenin pathway. In the absence of WNT ligands, the transmembrane GPCR complex made up of Frizzled (FZD) and Low-density Lipoprotein receptor-related protein 5/6 co-receptors (LRP5/6) is inactivated preventing β-catenin destruction complex recruitment (AXIN, APC, CK1α, and GSK-β) [122]. This leads to the N-terminus phosphorylation of β-catenin by CK1α and GSK-3β and its proteolysis. Upon binding of WNT ligands to FZD and LRP5/6, dishevelled (DVL) is phosphorylated and binds to AXIN, resulting in inhibition of the β-catenin destruction complex recruitment [122] (Fig. 5). The recruitment of the complex prevents β-catenin proteolysis, and unphosphorylated β-catenin moves into the nucleus due to increasing cytoplasmic concentrations and binds with TCF/LEF to activate transcription of WNT target genes such as *CCND1*, *C-MYC*, *MMP-7* and *JAGGED* (Fig. 5).

In synovial sarcoma, high levels of nuclear staining of β-catenin are common in patient samples and are associated with decreased survival and increased lung metastasis [123]. The SS18::SSX fusion acts upstream of the WNT signalling pathway to induce β-catenin nuclear localisation, inhibition of transcriptional repressors TLE and HDAC, as well as binding to the TCF/LEF transcription complex [124]. Furthermore, the stabilisation of β-catenin also contributes to SS18::SSX-driven synovial sarcoma, by increasing the transformation of mesenchymal progenitors, enhancing tumorigenesis [124]. Inhibition of the WNT/β-catenin pathway using radioimmunotherapy compounds targeting FZD significantly decreased tumour volume in Balb/c nude mice with negligible toxicity [125].

In Ewing sarcoma, Cadherin-11 (*CDH11*) and *LGR5* have been demonstrated to enhance WNT/β-catenin signalling through stabilisation of β-catenin [126]. Therapeutically, inhibition of the WNT/β-catenin pathway in Ewing sarcoma with the PORCN inhibitor, WNT974, decreases metastatic Ewing sarcoma in a mouse model but does not affect overall survival [127]. Increased β-catenin expression is also linked to increased metastasis, shorter overall survival periods and increased EMT in osteosarcoma [128, 129]. Pathway inhibition using the PORCN inhibitor ETC-159 decreased WNT signalling, increased apoptosis *in vitro* and increased tumour necrosis *in vivo* [130]. Furthermore, TANKYRASE silencing through siRNA and pharmacological agents results in reduced osteosarcoma cell growth, increased apoptosis, and has a synergistic interaction with doxorubicin to decrease tumour progression *in vivo* [131]*.*

### Hippo signalling

**Fig. 6:**
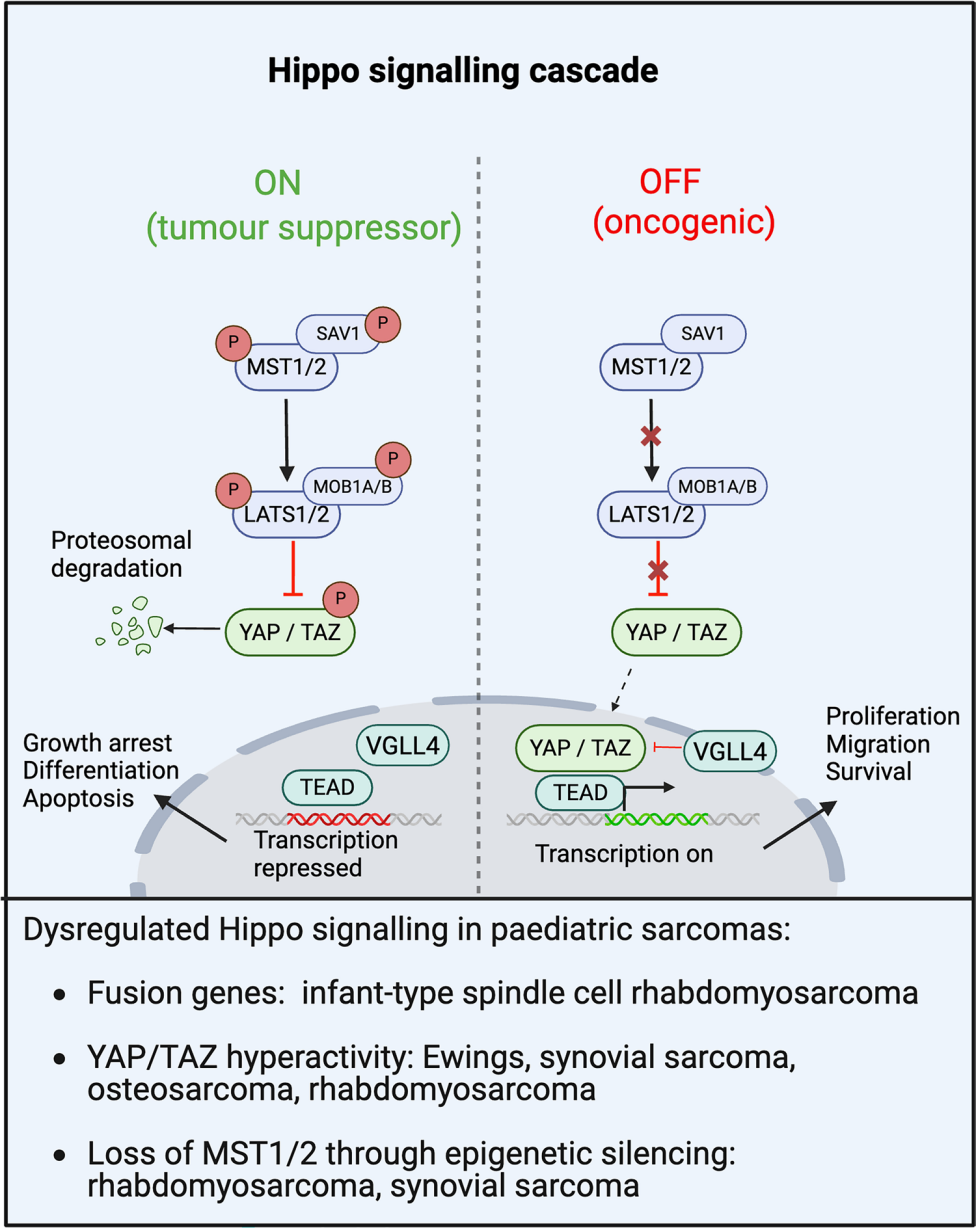
Hippo signalling pathway in paediatric sarcomas. In response to upstream stimuli which activate the pathway, MST1 and MST2 phosphorylate and activate LATS1 and LATS2. Protein Salvador homolog 1 (SAV1) and MOB kinase activator 1a/b (MOB1A/B) act as scaffold proteins for MST1/2 and LATS1/2 respectively. LATS1 and LATS2 then phosphorylate the amino acid motifs of transcriptional cofactors YAP and its paralog TAZ, leading to their proteosomal degradation in the cytosol. When the pathway is inactivated, the upstream inhibition of YAP and TAZ is Lost, and active unphosphorylated intranuclear YAP and TAZ bind to a variety of transcription factors, predominantly transcriptionally enhanced associated domain 1–4 (TEAD1-4) proteins to mediate gene expression. Vestigial-like proteins, particularly VGLL4, selectively compete with YAP for TEAD binding; thus, they can antagonise YAP. Oncogenic functioning of this pathway can arise either from interruption of upstream inhibitory components such as MST1/2 or LATS1/2 or from hyperactivity of the downstream effector proteins YAP, TAZ and TEAD. There is extensive crosstalk with other oncogenic signalling pathways which can independently activate YAP/TAZ

The Hippo signalling pathway is an evolutionarily conserved developmental pathway implicated in organogenesis, metabolism, tissue homeostasis, proliferation, and stem cell renewal [132]. When activated (Fig. 6), the Hippo signalling cascade has tumour suppressive functions, inhibiting proliferation and promoting differentiation and apoptosis through negatively regulating the transcription of genes involved in cell division, migration, and pro-survival signalling.

In response to upstream stimuli, Mammalian sterile 20-like kinases 1 and 2 (MST1 and MST2) phosphorylate and thus activate the large tumour suppressive kinases 1 and 2 (LATS1 and LATS2) [133]. LATS1 and LATS2 then phosphorylate the amino acid motifs of transcriptional cofactors YES1-Associated protein (YAP) and its paralog PDZ-binding motif TAZ, leading to proteasomal degradation of YAP/TAZ in the cytosol [133, 134]. When the pathway is inactivated, the upstream inhibition of YAP and TAZ is lost, and active intranuclear YAP and TAZ bind to a variety of transcription factors, predominantly transcriptionally enhanced associated domain 1–4 (TEAD1-4) proteins to mediate gene expression (132, 134). Vestigial-like proteins (VGLLs), particularly VGLL4, selectively compete with YAP for TEAD binding, thus can antagonise YAP-dependent growth [134, 135]. Other transcription factors targeted by YAP/TAZ include Erb-b2 receptor kinase 4 (ERBB4), T-box transcription factor 5 (TBX5), RUNX-family transcription factors 1–3 (RUNX1-3), and SMAD family members [136–139]. Furthermore, there is extensive crosstalk with other oncogenic signalling pathways which also regulate the expression of YAP and TAZ including WNT signalling, PI3K/AKT signalling, TGF-β, and G-protein coupled receptors [134, 140, 141]. Oncogenic functioning of this pathway can arise either from interruption of upstream inhibitory components such as MST1/2 or LATS1/2 (which function as tumour suppressors), or from hyperactivity of the downstream effector proteins YAP, TAZ and TEAD (which can function as oncogenes) (Fig. 6).

There is an abundance of evidence to suggest that dysregulation of Hippo signalling is recurrently implicated in sarcomagenesis, and this is reviewed in more detail by Salguero-Aranda *et al.* and Mohamed *et al.* [134, 142]. Immunohistochemical analyses of human sarcoma tissues demonstrate nuclear overexpression of YAP or TAZ in 53% and 33% of samples respectively [143], and loss of MST1, MST2 and LATS1 in 47%, 26% and 19% respectively [144]. Furthermore, YAP or TAZ expression has been reported to be an independent predictor of poor outcome in osteosarcoma [145], and knockout of YAP1 inhibits proliferation in osteosarcoma cells *in vitro* and *in vivo* [146]. Transgenic murine models also provide insight that genetic perturbations in Hippo components can produce tumours that histologically and transcriptionally recapitulate human sarcomas. For example, selective YAP hyperactivity due to the activating mutation *YAP1* S127A in satellite cells is sufficient alone to cause embryonal rhabdomyosarcoma [147]. Similarly, mice with homozygous disruption of *LATS1* develop ovarian stromal tumours and skin fibrosarcomas [148].

Curiously, despite strong evidence supporting the role of the Hippo pathway in sarcomagenesis, mutations or fusions of the genes directly involved in Hippo signalling are incredibly scarce [134]. There are only two sarcoma types that recurrently harbour alterations of Hippo genes, and both are very rare entities. Firstly, epithelioid haemangioendothelioma, which predominantly affects older adults, is driven by a fusion of WWTR1::CAMTA1 or YAP1::TFE3 in almost all cases [149, 150]. Secondly, the infant/congenital subtype of spindle cell rhabdomyosarcoma harbours fusions of VGLL2::NCOA2 or VGLL2::CITED, which occur in up to two-thirds of cases, and rarer TEAD1::NCOA2 fusions have also been reported [19].

Given the scarcity of genetic alterations in Hippo components in paediatric sarcomas, it is likely that significant crosstalk with other oncogenic signalling pathways or epigenetic factors mediates oncogenic hijacking of Hippo signalling. In alveolar rhabdomyosarcoma cells, the *PAX3::FOXO1 *fusion transcript has been demonstrated to suppress Hippo signalling through Ras associated domain family 4 (RASSF4) which modulates the activity of the MST1 kinase, leading to overexpression of YAP [151]. Similarly, functional studies in synovial sarcoma cell lines have linked the expression of the *SS18::SSX* fusion transcript to levels of YAP/TAZ, which have been mechanistically linked to IGF-1R/PI3K/AKT signalling [152]. Furthermore, in murine models, Hedgehog signalling induces osteosarcoma development through YAP overexpression, and inhibition of Hedgehog signalling reduces YAP expression [153]. These examples highlight that Hippo-independent mechanisms can regulate the downstream effector components of the Hippo pathway. Furthermore, MST1 is methylated in 80% of rhabdomyosarcoma, and MST2 is methylated in 50% of synovial sarcoma [154]. This suggests that epigenetic silencing of upstream inhibitory kinases is another mechanism by which sarcomas modulate Hippo signalling in the absence of Hippo gene mutations.

Current options for targeting the Hippo pathway are limited. The only direct inhibitor of YAP/TAZ is verteporfin, which prevents binding to TEAD. Verteporfin has been shown to reduce proliferation of Ewing sarcoma, synovial sarcoma and rhabdomyosarcoma cell lines [152, 155, 156]. Although no clinical trials have been performed to investigate verteporfin in sarcoma, early-phase trials are underway for some other cancer types such as glioblastoma (NCT04590664). Furthermore, statins appear to indirectly reduce the expression of YAP/TAZ through modulating the mevalonate pathway [157] and are under investigation in a phase I clinical trial for relapsed or refractory paediatric solid tumours in combination with cyclophosphamide and topotecan [158]. Finally, several multi-kinase inhibitors, including pazopanib and dasatinib, have been shown to indirectly inhibit YAP/TAZ [157]. The utility of multi-kinase inhibitors in paediatric sarcoma is discussed in the next section.

## Protein kinase signalling

Protein kinases govern protein activity, expression and localisation within the cell and function in numerous signalling cascades and essential cellular processes required for development and tissue homeostasis. There are at least 538 protein kinases encoded by the human genome, the full complement of which are collectively referred to as the kinome [159]. Protein kinases are enzymes that transfer γ-phosphate groups from ATP to serine, threonine or tyrosine residues on their target protein, turning a protein ‘on’, and usually function to promote cell proliferation, survival, and migration [159]. Comparatively, phosphatases function opposingly to remove the phosphate group turning the protein and its signalling pathway ‘off’ [159]. Together, these two enzymatic processes enhance the plasticity of the epigenome by providing another layer of control over protein function throughout the cell. The kinase-phosphatase balance is responsible for committing cells to a specific developmental pathway and plays crucial roles in stem-cell renewal, embryogenesis, differentiation, and tissue expansion [160]. However, dysregulation of this balance, such as through constitutive activation of protein kinases, can shift the kinome of a cell into an oncogenic state, and this is frequently implicated in sarcoma development, metastasis, progression, and therapy resistance [159, 161]. The mechanisms that lead to constitutive activation of a protein kinase can include gene fusions that create chimeric receptor kinases, gain-of-function point mutations, overexpression due to gene amplification or epigenomic dysregulation, or autocrine signalling [162].

**Fig. 7:**
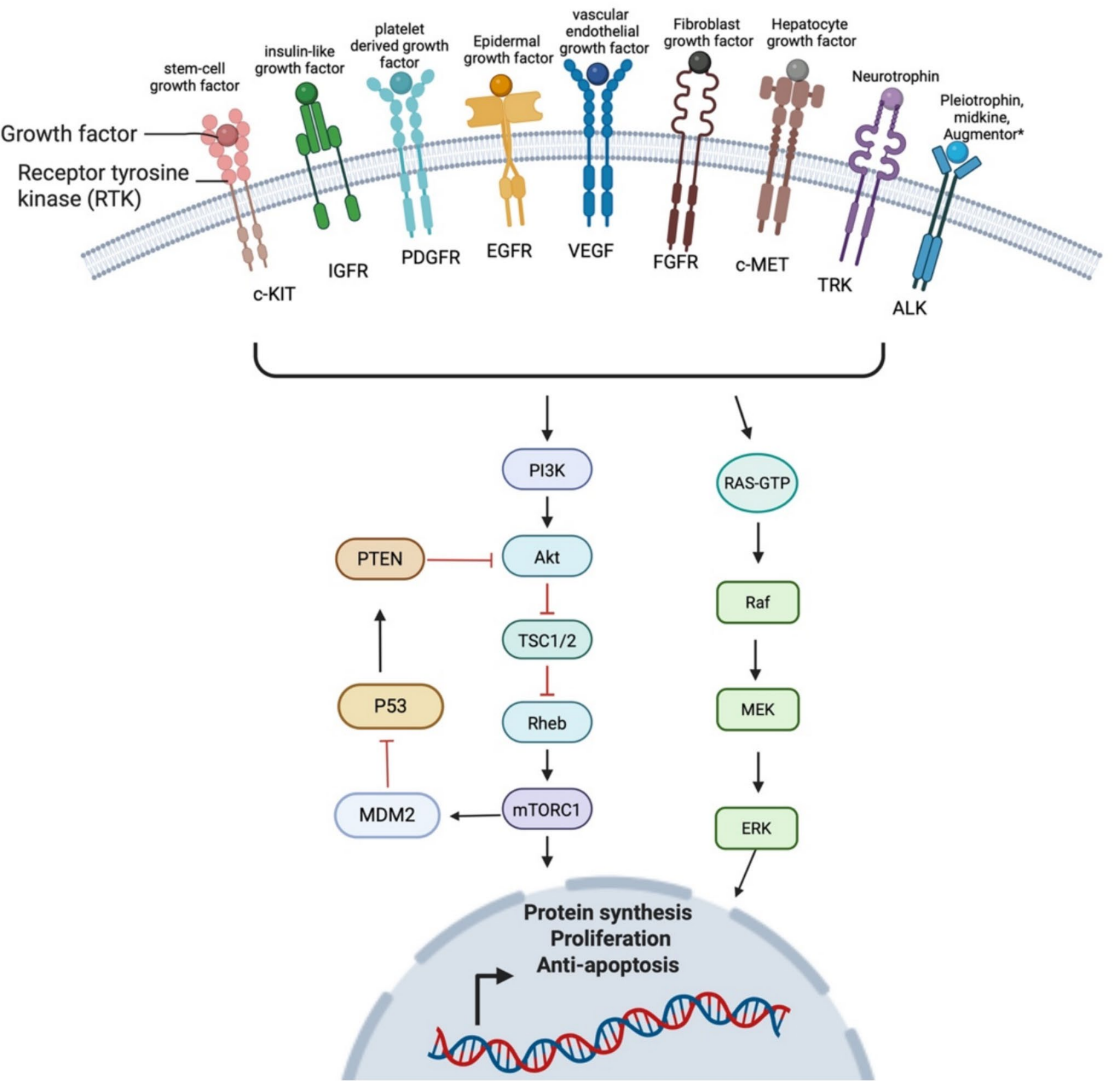
Kinase signalling pathways relevant to childhood sarcomas. Receptor kinases are activated by ligand growth factors, triggering downstream signalling cascades through common pathways such as RAS/MEK/ERK and PI3K/AKT/mTOR which promotes proliferation and inhibits P53-mediated apoptosis. Receptor kinases may become constitutively active in oncogenesis due to fusions, point mutations, amplification, epigenetic mechanisms or autocrine signalling, leading to sustained and inappropriate cell growth and apoptotic silencing. *The ligands for the ALK receptor are still under investigation

Serine/threonine kinases (STKs) and tyrosine kinases (TKs) are the two largest protein kinase families and are heavily implicated in both embryological development and oncogenesis. These two kinase families can further be divided into receptor kinases, which are usually located on the cell surface and are receptors for extracellular triggers such as growth factors, and non-receptor kinases, which are located in the cytoplasm and act downstream in intracellular signalling cascades in response to receptor kinase activation or other sources [159, 162] (Fig. 7).

Receptor tyrosine kinases that are frequently implicated in sarcomagenesis include platelet-derived growth factor receptor (PDGFR-α and PDGFR-β), epidermal growth factor receptor (EGFR), the stem cell factor receptor known as c-Kit (KIT), insulin-like growth factor receptor (IGFR), hepatocyte growth factor receptor c-MET, and vascular endothelial growth factor receptor (VEGFR) [163]. Serine/threonine receptor kinases include TGF-β receptors. When activated by binding of their specific growth factor ligand, these receptor kinases trigger downstream growth and pro-survival signalling through non-receptor serine/threonine kinases including mitogen-activated protein kinase (MAPK), cyclin-dependent kinases (CDKs), RAC-α serine/threonine protein kinase (AKT), and mammalian target of rapamycin (mTOR) amongst others, and the non-receptor tyrosine kinase Janus kinase (JAK) [162]. These typically function in intracellular signalling cascades and in the regulation of cell cycle and growth [162] (Fig. 7).

### Receptor kinase signalling

PDGFR-α and -β, c-KIT and VEGFR2 are some of the most frequently overexpressed receptor tyrosine kinases in paediatric sarcomas [164]. In a study of 96 paediatric non-rhabdomyosarcoma soft tissue sarcomas, overexpression of PDGFR-α, PDGFR-β, VEGFR2 and c-KIT occurred in 32.3%, 17.7%, 19.8%, and 8.3% respectively [164]. In another study that was not paediatric-specific, synovial sarcoma tissue demonstrated high expression of PDGFR-α ligands and receptor, and overexpression was associated with metastasis and relapse [165]. Furthermore, genomic sequencing of 71 paediatric and adult osteosarcoma samples reported 20% had an amplification at 4q12 spanning *PDGFR*A,* VEGFR2*, and *KIT*, and in total, 40% of participants had either *PDGFRA* or *VEGF* amplification [166]. Overexpression of these pathways has been linked to metastasis in osteosarcoma, but the evidence is conflicting regarding whether their expression levels in tumour tissue have prognostic implications [167]. In Ewing sarcoma xenograft models, inhibiting PDGFR-β reduces growth and metastasis [168]. IGF signalling is also implicated in pathogenesis, metastasis and treatment resistance in Ewing sarcoma [169, 170]. The IGF-1 receptor is ubiquitously expressed in Ewing tissue and is activated by autocrine production of IGF1 by the malignant cells [171, 172]. Furthermore, IGFs are also implicated in rhabdomyosarcoma, osteosarcoma and synovial sarcoma, and targeting IGFR *in vitro* with antibodies results in apoptosis and inhibits growth [173].

### Downstream signalling cascades

The occupancy of receptor kinases with growth factor ligand results in the activation of numerous downstream signalling cascades, in particular the PI3K/AKT/mTOR pathway and the Ras/Raf/MEK/ERK pathway, also known as the MAPK pathway. *PIK3CA* mutations occur at relatively low frequency in most paediatric sarcomas; however, the PI3K/AKT/mTOR pathway is frequently dysregulated in many paediatric sarcoma types through other mechanisms such as excessive activity of upstream receptor kinases and loss of the negative regulator PTEN, which is silenced in 38% of sarcomas [107]. This is demonstrated well by the fact that only 5.4% of rhabdomyosarcomas have an activating mutation in the *PIK3CA* gene, but whole-genome, whole-exome and transcriptome sequencing on 44 rhabdomyosarcoma samples identified dysregulation of the PI3K/AKT/mTOR pathway by upstream receptor kinase regulators in 93% of samples [16]. Furthermore, the PI3K/AKT/mTOR pathway is also implicated in chemotherapy resistance. For example, treatment of synovial sarcoma cells with the PI3K-inhibitor LY294001 inhibited synovial sarcoma proliferation and synergised with cytotoxic agents *in vitro* [174]. The MAPK/ERK pathway is another downstream effector pathway frequently altered in childhood sarcomas, such as osteosarcoma, rhabdomyosarcoma and Ewing sarcoma, where its activation promotes metastasis and migration, and inhibition results in cell death [175]. Mutations in MAPK pathway genes are the most common class of genetic alteration identified in embryonal rhabdomyosarcoma, occurring in up to 42% [17]. These include activating mutations in positive regulators of MAPK signalling such as *KRAS*,* NRAS* and *HRAS*, or deletion/inactivation of the negative regulator *NF1* (Table 1) [16]. Furthermore, there is a higher proportion of MAPK/RAS gene alterations reported in high-risk embryonal rhabdomyosarcoma, with 75% (6/8) high-risk tumours harbouring RAS pathway alterations, compared with 0% (0/10) of low-risk disease in one study [176]. In patient-derived murine models of RAS-mutated rhabdomyosarcoma, the MEK inhibitor trametinib results in a modest reduction in tumour growth, which is further enhanced when combined with the synergistic IGF1-R inhibitor gatinutmab [177].

### Targeting protein kinases

Protein kinases provide an attractive target in treating childhood sarcoma due to the ‘druggable’ nature of the molecules and the striking success of tyrosine kinase inhibitors in other malignancies such as chronic myeloid leukaemia (CML) or gastrointestinal stromal tumours (GIST) [159]. However, inefficacy or resistance to kinase inhibitors may occur due to compensatory upregulation of alternative signalling pathways, activation of a pathway downstream from where the drug acts, or mutations within the kinase which render them unresponsive to inhibitors [159]. Despite protein kinases clearly being implicated in the pathogenesis of childhood sarcomas, and kinase inhibitors showing promising activity in preclinical models, successful translation into the clinic for most sarcomas has been limited and remains an ongoing area of investigation. Although it is beyond the scope of this review article to discuss each protein kinase inhibitor in detail, a few select examples relevant to paediatric sarcoma are discussed below, and in more depth by Kyriazoglou et al., Fleuren et al., and Wilding et al. [163, 178, 179].

Pazopanib, a multi-kinase angiogenesis inhibitor targeting c-Kit, VEGFR-1 and VEGFR-2, PDGFR-α and -β, was the first tyrosine kinase inhibitor approved by the FDA for the treatment of refractory soft tissue sarcomas in 2012, based on results of the phase III randomized double-blinded placebo-controlled PALETTE trial that demonstrated improved progression-free survival but not overall survival in adults with advanced non-adipocytic soft tissue sarcoma [180]. A subsequent randomised controlled trial, ARST1321, investigated pazopanib in 85 children and adults with non-rhabdomyosarcoma STS having neoadjuvant chemotherapy prior to surgery and found significantly improved histological response (defined as > 90% tumour necrosis) in the pazopanib arm (58.3%) vs the control arm (22.2%) [181] but found no differences in outcome [182]. Similarly, in bone sarcomas, levantanib, a multi-kinase inhibitor targeting VEGF, FGFR, PDGFR-α, and KIT showed promising preclinical activity in osteosarcoma. However, in the ITCC-050 study, which included 31 paediatric and young adult patients with relapsed or refractory osteosarcoma, only two patients had a partial response (ORR 6%), all patients experienced adverse effects, and 20/31 experienced grade 3 or greater adverse effects [183].

Many trials are now investigating multi-kinase inhibitors in conjunction with other targeted molecular therapies or conventional cytotoxic agents in an effort to increase the efficacy of these drugs and decrease the development of resistance. Early-phase clinical trials of IGF-1R-targeted antibodies in Ewing sarcoma reported a partial response in 10–14% of patients, but responses were short-lived [184]. A meta-analysis of 5 clinical trials encompassing 53 patients with Ewing sarcoma treated with IGF-1R-targeted antibodies in combination with or without mTOR inhibitors reported a two-fold increase in progression-free survival with the two-combination compared to IGF-1R antibodies alone (PFS 1.6 vs 3.3 months, *p* = 0.042) [179, 184]. A phase III Children’s Oncology Group randomized controlled trial of the IGF-1R antibody ganitumab in combination with standard of care chemotherapy for children with metastatic Ewing sarcoma unfortunately showed no benefit in event-free survival and increased toxicity in the experimental arm [185]. Similarly, in children with intermediate-risk rhabdomyosarcoma randomized to receive standard of care chemotherapy with or without the mTOR inhibitor temsirolimus, no difference in event-free survival was observed in the ARST1431 study [186].

Regorafenib, which targets RET, PDGFR-β, KIT and VEGFRs, in adult patients with advanced Ewing sarcoma demonstrated short-term benefit with 56% of patients in the treatment arm vs 7.7% of patients in the placebo arm progression-free at 8 weeks, with a median PFS of 11.4 vs 3.9 weeks when used as monotherapy [187]. Regorafenib is currently in phase III clinical trial in paediatric patients with Ewing sarcoma in conjunction with standard of care therapy (ACTRN12624000532505). Similarly, cabozantinib, another multi-kinase inhibitor which targets MET, RET, AXL, VEGFR-2, and c-KIT, resulted in 6-month disease stability in 33% of relapsed osteosarcoma patients as monotherapy [188], and is now in early-phase randomised clinical trials in conjunction with standard of care chemotherapy (NCT05691478).

There are, however, two very rare paediatric sarcoma types that have recently shown marked success with specific kinase inhibitors as monotherapy. Infantile fibrosarcoma is a previously devastating sarcoma that affects very young infants and is congenital in 50% of cases [189]. Infantile fibrosarcoma is almost exclusively driven by activating fusions of *NTRK* that encode a constitutively active mutant TRK receptor, with 85% harbouring ETV6-NTRK3 and the remainder usually demonstrating novel NTRK-variant fusions [189]. The ligand for various TRK receptors, neurotrophins, is a growth factor that regulates neuronal migration and differentiation in the vertebrate nervous system [190]. These tumours have pronounced and durable responses to NTRK inhibitor larotrectinib monotherapy, which has minimal toxicity and only a 7.8% treatment failure rate compared to 35% treatment failure with previous standard of care therapy including chemotherapy, surgery and/or radiation [191]. Furthermore, activating fusions of NTRKs occur extremely rarely in other cancers, including the common sarcoma types, but when present, are relevant as they represent a druggable target that is considered tumour agnostic [35]. Similarly, inflammatory myofibroblast tumours (IMTs) are an extremely rare intermediate-grade mesenchymal tumour that rarely metastasises but is locally invasive and predominantly occurs in young children [192]. Paediatric IMTs harbour activating fusions of anaplastic lymphoma kinase (ALK) in > 90% and are highly responsive to treatment with ALK-inhibitor as monotherapy [192].

## Epithelial to mesenchymal transition and related processes

In early embryogenesis, epithelial cells demonstrate marked phenotypic plasticity and can reversibly develop mesenchymal properties to allow for tissue migration and invasion, in a process termed epithelial to mesenchymal transition (EMT) [193]. This process is implicated in many developmental processes, including neural crest formation and migration, organ development, and enables the trophoblast to invade the placental tissue [194]. Epithelial cells have a propensity to exist in highly organised and cohesive cell populations, characterized by tight adherence to basement membrane and neighbouring cells due to E-cadherin, integrins and intracellular junctions enforced by apical-basal polarity [195]. EMT allows epithelial cells to escape these rigid constraints by assuming a mesenchymal phenotype with reduced cell–cell adhesions, front-back polarity (which facilitates migration), invasion of the basement membrane, cell individualisation, and cytoskeletal remodelling resulting in a change in morphology to a fibroblast-like spindle cell appearance [195]. In addition to the critical role this phenomenon plays in development and a diverse range of physiological functions, EMT also drives oncogenesis and metastasis. EMT is a well-established mechanism by which carcinomas can reversibly assume mesenchymal features to facilitate distant metastasis, intravascular migration, tissue invasion, and chemoresistance. Once established at a distant site, the cancer cells revert to their epithelial phenotype, through a process of mesenchymal to epithelial transition (MET), to resume their proliferative, organised and adherent epithelial qualities to establish a metastasis [195]. At a molecular level, EMT is driven by loss of the adhesion molecule E-cadherin, activation of the transforming growth factor β (TGF-β) pathway, and upregulation of organogenesis transcription factors such as twist-related protein 1 (TWIST-1), SNAIL (SNAI1), SLUG (SNAI2) and Zinc finger E-box-binding homebox (ZEB) 1 and 2 [196].

Interestingly, there is emerging evidence that sarcomas undergo a similar dynamic plasticity of phenotype that is reminiscent of EMT and MET, even though this seems paradoxical, as they are, by definition, a mesenchymal malignancy. It has been proposed that the mesenchymal properties of sarcoma cells perhaps explain their aggressive nature and the propensity for early metastasis to distant sites via hematogenous spread. However, EMT/MET can be considered a spectrum, and although sarcomas may not undergo the complete spectrum, sarcomas still appear to upregulate pathways that promote mesenchymal features (EMT pathways) to enhance their migratory capacity and, similarly, undergo MET, developing epithelial-like properties to establish metastasis [195, 197].

A meta-analysis of 812 paediatric and adult bone and soft tissue sarcoma across 8 different studies found that low expression of E-cadherin was associated with poorer outcomes and higher-stage disease [198]. Furthermore, in paediatric rhabdomyosarcoma cohorts, epigenetic overexpression of guanine nucleotide exchange factor T (GEFT), due to promoter methylation, has been associated with a poor prognosis and metastatic disease. The oncogenic function of GEFT in rhabdomyosarcoma cell lines and mouse models has been demonstrated to be due to overexpression of EMT markers N-Cadherin, SNAIL, TWIST, SLUG and ZEB [199]. Furthermore, treatment of osteosarcoma cell lines with TGF-β has been shown to cause loss of E-cadherin expression and upregulation of EMT markers, resulting in increased cell migration [197]. In synovial sarcoma, a similar phenomenon is observed where TGF-β drives loss of epithelial characteristics [200] and facilitates migration, while the SYTSSX1 or 2 fusion transcript drives MET through binding and inactivating SLUG and SNAIL [201].

## Emerging therapies and future directions

No significant change in standard of care therapies since last century has resulted in a stagnation of survival rates for childhood sarcomas and highlights the urgent need for new therapeutic approaches. This has in part been hindered by the rarity and complex heterogeneity of many sarcoma types. With advances in next-generation sequencing technology in the last two decades, our understanding of the genetic and molecular characteristics of sarcomas is rapidly increasing and has paved the way for more personalized or precision-based clinical management opportunities that enable patients to be matched and treated with therapies that they are most likely to benefit from. As a result, biomarker-driven clinical trials are becoming more prevalent and there are increasing numbers of national precision-based molecular profiling clinical trials available to sarcoma patients, including the ZERO Childhood Cancer Program and the MoST Study, based in Australia [202, 203].

**Fig. 8:**
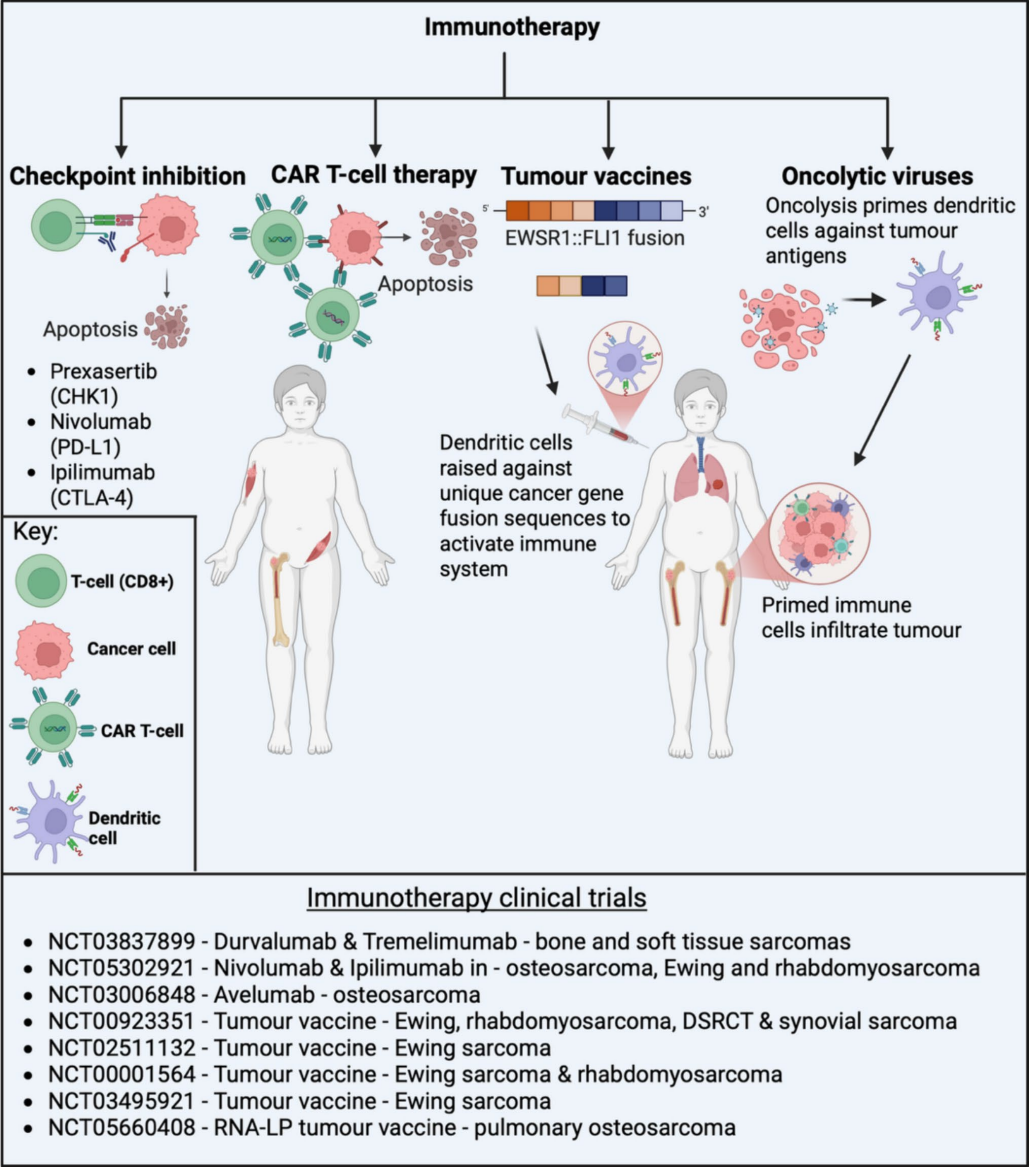
Immunotherapies in development for paediatric sarcoma. Overview of the types of immunotherapies being investigated in paediatric sarcoma and their mechanism of action. Checkpoint inhibition utilises normal immune system functions to target cancer cells by inhibiting receptors such as PD-L1, causing the cancer cells to appear foreign and triggering an immune response. CAR T-cell therapy utilises T cells from a patient that are raised against tumour antigens *ex vivo* to generate a rapid anti-tumour response against any cell expressing the target antigen of interest when administered back to the patient. This process circumvents potential immune-suppressive features of the cancers while increasing specificity compared to checkpoint blockade. Tumour vaccines further stimulate potent immune responses against cancer cells through specific targeting of unique proteins that present on the tumour cell surface (e.g. EWSR1::FLI1 fusion peptide). Oncolytic viruses infect cancer cells, replicate and lyse, which results in antigen presenting dendritic cells to express cancer specific antigens on their cell surface stimulating CD-8 + cytotoxic T cells to also attack the tumour

**Fig. 9:**
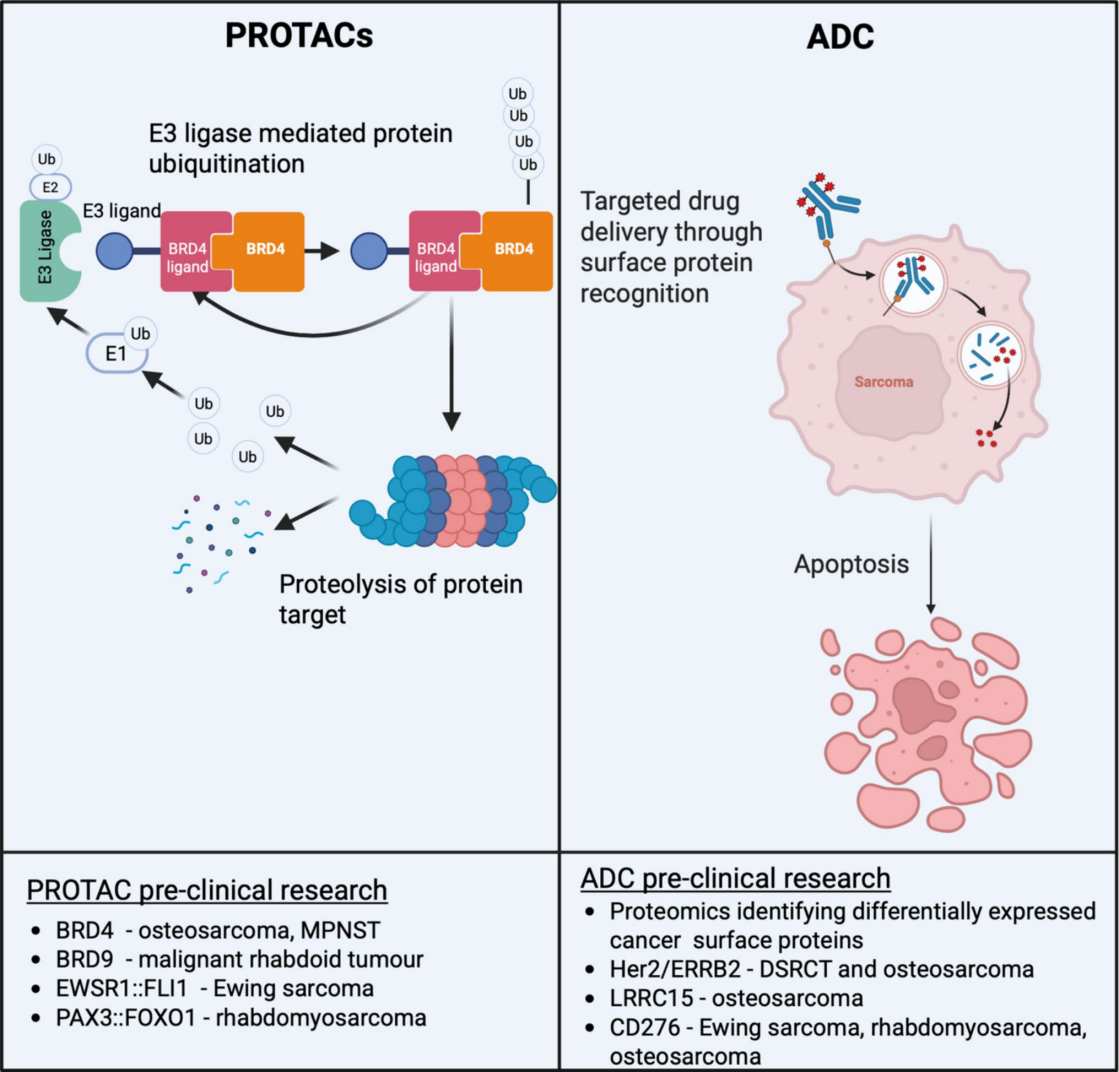
Emerging pre-clinical treatments of paediatric sarcoma. PROTACs are a unique ubiquitin-proteasome system that can be used to degrade specific proteins. In the context of sarcoma, BRD4, BRD9, EWSR1::FLI1 and PAX3::FOXO1 are proteins that have been currently investigated as potential targets of PROTAC therapy. These drugs function via a protein recognising ligand coupled to an E3 ligase ligand that enables ubiquitination of the target protein, resulting in its proteolysis. In contrast, ADCs function through internalisation of antibodies conjugated to a drug of interest, such as monomethyl auristatin E. This system enables targeted delivery with drug release within the cell following degradation of the antibody following internalisation

Targeted molecular therapies that inhibit key underlying dysregulated developmental and oncogenic pathways in sarcoma represent an important therapeutic opportunity, and many examples of these have been discussed above. However, the genomic complexity of most sarcomas, and the ability of tumours to adapt to treatment and develop resistance, suggests that one treatment modality alone is unlikely to result in sustained response. Accordingly, current, new and complementary treatment modalities will be needed in different combinations and at different stages of a patient’s cancer journey, including chemotherapy, radiotherapy, targeted molecular therapies, continually evolving immunotherapies (Fig. 8), and emerging proteolysis-targeting chimera (PROTAC) and antibody-drug conjugate (ADC) therapies (Fig. 9). While standard-of-care chemotherapy and radiotherapy, and many targeted therapies for sarcoma have already been discussed above and are reviewed extensively in the literature, we are only at the beginning of realising the potential of emerging therapies such as immunotherapy, PROTACs and ADCs.

### Immunotherapy

Checkpoint blockade and chimeric antigen receptor T-cell (CAR T-cell) therapy are amongst the most investigated immunotherapies in the treatment of paediatric sarcoma (Fig. 8). The PD-L1 inhibitor atezolizumab was recently shown to be effective in children and adults with advanced alveolar soft part sarcoma (ASPS) with a response rate of 37% and is now FDA approved for this indication [204]. Unfortunately, there has been less success so far with checkpoint blockade in the more common childhood sarcomas. Prexasertib, a CHK1 inhibitor, has been demonstrated to elicit a strong anti-tumour response in preclinical models of DSRCT, malignant rhabdoid tumour, and rhabdomyosarcoma but variable responses in Ewing sarcoma and osteosarcoma [205]. The response was enhanced when combined with irinotecan and doxorubicin [205]. Nivolumab, a PD-L1 inhibitor, with or without the CTLA-4 inhibitor ipilimumab, proved ineffective in osteosarcoma, Ewing sarcoma, synovial sarcoma and DSRCT in the ADVL1412 trial. The lack of efficacy is likely related to low PD-L1 levels in the sarcomas analysed with the majority showing no expression [206], highlighting the importance of biomarker-driven clinical management. Another clinical trial investigating ipilimumab in 33 paediatric patients with advanced solid tumours showed that 4 of the 17 enrolled sarcoma patients achieved stable disease, but broad endpoint outcomes such as overall survival were not investigated. The limited efficacy of checkpoint inhibitors against osteosarcoma and Ewing sarcoma could be attributed to low checkpoint receptor and ligand expression.

T-cell-based therapies for paediatric sarcomas are an area of ongoing research. In rhabdomyosarcoma PDX models, CD276 targeting CAR T-cells (CD276.8HTM.BBz) significantly decreased tumour volume [207]. CAR.GD2 T-cells cause reduced viability in osteosarcoma and alveolar rhabdomyosarcoma cell lines and decrease tumour volume in embryonal rhabdomyosarcoma xenograft models [208]. Comparatively, GD2-directed CAR T-cells potentiated the effects of an HGF-neutralizing antibody, AMG102, in Ewing sarcoma cell lines [209]. Promisingly, a T-cell receptor therapy for synovial sarcoma has recently been successfully translated into the clinic. In the SPEARHEAD-1 trial, 44 heavily pretreated adults with advanced synovial sarcoma who had the HLA-A*02 genotype and had tumour expression of melanoma-associated antigen 4 (MAGE-A4) received a single dose of afamitresgene autoleukcal, a genetically modified autologous T-cell receptor immunotherapy targeting MAGE-A4, resulting in a 39% response rate with a median duration of response of 6 months [210]. This therapy is now FDA approved in adults and is under investigation in children with synovial sarcoma, MPNST and osteosarcoma (NCT05642455) but is limited to MAGE-A4 expressing tumours and HLA-eligible patients.

Since checkpoint blockade and CAR T-cell therapies rely on immune cell function, the immunologically cold nature of paediatric sarcoma makes them less effective than in other cancer types. Therefore, potential therapeutic strategies that elicit an immune response against cancer, such as tumour vaccines and oncolytic viruses, are being explored (Fig. 8). One such clinical trial used a dendritic cell (DC) vaccine designed to recognise peptides relating to fusion proteins such as those in Ewing sarcoma and rhabdomyosarcoma. This study enrolled newly diagnosed metastatic or recurrent high-risk patients with Ewing sarcoma (*n* = 20/*n* = 4: immunotherapy/no immunotherapy), rhabdomyosarcoma (*n* = 6/*n* = 5), DSRCT (*n* = 2/*n* = 3) and synovial sarcoma (*n* = 1/*n* = 1); however, only the Ewing sarcoma and rhabdomyosarcoma patients showed benefit, with a 5-year overall survival of 63% compared to 0% for the other patient cohorts [211]. Other studies have reported shorter and less pronounced responses [212]. Immunosuppression due to the amount of pre-treatment chemotherapy and radiotherapy and the ratio of cytotoxic T-cells to Tregs are suggested to be potential indicators of response [213]. Mixed outcomes have also been reported for oncolytic viruses, with preclinical studies showing synergistic effects when combined with other therapies such as T-cell therapies and trabectedin [214] whereas viral monotherapy resulted in increased cell cycle arrest and apoptosis but poor survival outcomes *in vivo* [215]. Interestingly, pre-conditioning of CAR-T cells with a CDK4/6 inhibitor increased the anti-tumour effect of oncolytic viruses in a mouse model of Ewing sarcoma [216]. Despite the limited and variable responses of sarcomas to immunotherapy, it remains an area of intense research, and with a better understanding of biomarkers predictive of response and optimal combination with existing therapies, it could provide future avenues of improved treatment.

### Proteolysis-targeting chimera (PROTAC)

PROTACs are an emerging therapeutic in paediatric sarcoma and harness the ubiquitin-proteasome system to degrade target oncogenic proteins (Fig. 9). A recent review article by Mancarella et al. [217] summarises the targetable gene relationships in Ewing sarcoma, rhabdomyosarcoma, osteosarcoma, and synovial sarcoma in detail [217]. Furthermore, they provide a strong rationale for the use of PROTACs in targeting gene fusions in paediatric sarcoma such as EWSR1::FLI1 and PAX3::FOXO1 [217]. In osteosarcoma preclinical models, PROTACs that target BET family proteins, specifically BRD4, 3 and 2 synergise with chemotherapy, trigger apoptosis, cell cycle arrest and significantly reduce tumour volume [218, 219]. PROTACs targeting BRD4 and BRD9, components of the SWI/SNF (BAF) remodelling complex, have also shown efficacy in preclinical MPNST, malignant rhabdoid tumour and synovial sarcoma models [220–222]. The SMARCA4/SMARCA2 PROTAC, ACBI1, is also reported to have some efficacy in preclinical rhabdomyosarcoma models [223]. Thus, simple-karyotype sarcomas driven by oncogenic fusions or SWI/SNF mutations show promise for PROTAC therapy.

### Antibody-drug conjugates (ADC)

The ADC class of therapeutics is typically monoclonal antibodies against tumour-specific cell surface proteins, covalently attached to cytotoxic drugs enabling the delivery of cytotoxic payloads at high concentrations directly to the tumour cells to enhance efficacy and minimise off-target toxicity (Fig. 9). Surfaceome profiling studies are expanding databases for the identification of proteins that are highly expressed on sarcoma cells and are suitable for ADC design. Studies in osteosarcoma and rhabdomyosarcoma have identified 11 and 9 surface proteins, respectively, including FGFR4, MEGF10, CD276, AGRL2, GPC4, JAM3, CADM2, NCAM1, L1CAM, MT1-MMP, MRC2, CD276 and LRRC15 [224, 225]. In osteosarcoma PDX models, LRRC15-monomethyl auristatin (MMAE) conjugates significantly decreased tumour volume compared to cisplatin alone [226]. Furthermore, CD276-pyrrolobenzodiazepine increased event-free survival with exceptional responses in PDX models of embryonal and alveolar rhabdomyosarcoma and Ewing sarcoma [227]. Lastly, in DSRCT two HER2/ERRB2 ADCs, with trastuzumab deruxtecan and trastuzumab emtansine, significantly decreased cell viability and tumour growth and prolonged event-free survival [228].

Early-phase clinical trials of ADCs in sarcoma are currently underway. A phase II trial of lorvotuzumab mertansine (anti-CD56) in 62 children with relapsed solid tumours included 17 children with rhabdomyosarcoma and 10 with synovial sarcoma. There was one partial response and one complete response in a child with rhabdomyosarcoma and synovial sarcoma respectively and the treatment was well tolerated [229]. Although the use of ADCs in sarcoma is still in early stages, given the lack of targetable driver mutations, ADCs represent a promising area of further research.

## Conclusion

Developmental origins underpin childhood sarcoma initiation and progression, offering unique vulnerabilities that could be exploited for therapeutic targeting. The same mechanisms that are essential for human development also drive childhood sarcomagenesis when they inappropriately persist or re-activate in developing mesenchymal tissues. Oncogenic disruptions of the epigenetic machinery unleash embryonic chromatin landscapes and aberrant gene expression, driving migration, stemness and uncontrolled growth. Dysregulation of the classical embryological signalling pathways such as Hedgehog, Notch, Wnt/β-catenin, and Hippo results in a cell that favours self-renewal over differentiation. Constitutive activation of protein kinases leads to uncontrolled ligand-independent proliferation, and epithelial-mesenchymal plasticity permits metastasis and tissue invasion.

The molecular era of oncology has rapidly enhanced our understanding of the multi-omic landscape of childhood sarcomas, leading to the emergence of many promising novel therapeutic strategies. However, very few of these have been successfully translated into a clinical setting. Targeting the developmental pathways implicated in paediatric sarcomas has shown potential promise in preclinical models, and certain drugs, such as epigenetic therapies and multi-kinase inhibitors, are now under exploration in the clinic. Given children are still undergoing primary tissue development, caution must be used to ensure that these emerging therapies do not cause toxicity to post-natal tissue growth and development. Rigorous clinical trials are essential and must include long-term follow-up to monitor for adverse developmental side effects which could take years to become apparent.

Furthermore, modulation of underlying lineage-specific developmental and oncogenic pathways may reveal unrealised vulnerabilities. For example, differentiation therapies could induce protein expression changes that are targetable by PROTAC or ADC or drive a more permissive tumour microenvironment to enhance immunotherapy. Ongoing work to enhance our understanding of underlying tumour biology and the interplay of combinatorial treatments has the potential to unveil important therapeutic windows and opportunities. Given the unique biology and rarity of each childhood sarcoma type, collaborative international efforts are needed to translate these emerging strategies into clinically meaningful therapies and improve outcomes.

## Data Availability

No datasets were generated or analysed during the current study.
